# Synthesis and Antiallergic Activity of Dicoumarin Derivatives

**DOI:** 10.3390/molecules29163799

**Published:** 2024-08-10

**Authors:** Yuying Zhang, Xiaoyu Wang, Dejun Zhou

**Affiliations:** 1 The Key Laboratory of Chinese Medicine Research and Development in Hebei Province, Traditional Chinese Medicine Institute, Chengde Medical University, Chengde 067000, China; li00222i@163.com; 2 Experiment Center for Science and Technology, Shanghai University of Traditional Chinese Medicine, 1200 Cailun Road, Shanghai 201203, China

**Keywords:** dicoumarin, antiallergic, RBL-2H3 cells, mBMMCs

## Abstract

Allergies are one of the diseases whose incidence rates have increased in recent years due to the greenhouse effect and extreme climate change. Therefore, the development of new antiallergic drugs has attracted the interest of researchers in chemistry and pharmacy fields. Dicoumarin is a coumarin derivative with various biological activities, but its antiallergic activity has not been evaluated. In this study, 14 different dicoumarin derivatives were synthesized by diethylamine-catalyzed condensation reactions of 4-hydroxycoumarin with 14 different aldehydes, and they were identified on the basis of their spectral data. The dicoumarin derivatives were subjected to studies on the degranulation of rat basophilic leukemia cells (RBL-2H3 cells) and mouse bone-marrow-derived mast cells (mBMMCs), and some of them showed good inhibitory effects on the degranulation of the two types of mast cells, demonstrating their good antiallergic activity. This study presents a new method of developing new antiallergic drugs.

## 1. Introduction

Allergies, which can cause tissue damage or dysfunction, are disorders connected with the abnormal responses of the immune system to allergens [1]. According to the World Allergy Organization (WAO), nearly 40% of people in different parts of the world have experienced or are experiencing allergies [2]. The incidence rates of allergies in China have increased in recent years, with more than 200 million people suffering from these diseases. These diseases not only affect the quality of life of the patients but also place a heavy economic burden on the community [3]. Currently, corticosteroids and antihistamines are the main drugs for allergy treatment. However, they have some side effects, such as drowsiness, dry mouth, reduced vision, and lack of concentration [4]. Therefore, developing new antiallergic drugs with fewer side effects and high effectiveness in terms of the treatment of diverse allergies has become increasingly important. At present, there is no drug that cures allergies, excepting avoiding allergens. Clinically, the anti-allergy drugs used mainly include antihistamines (hydrochloride, loratadine, chlorphenamine, etc.); allergic reaction mediator blockers (montelukast sodium, ketotifen, cromolyn, tranilast, etc.); calcium, such as calcium gluconate and calcium chloride; immunosuppressants, such as corticosteroids (budesonide, dexamethasone, cyclophosphamide et al.); some Chinese traditional medicines for complementary treatment, such as Food Allergy Herbal Formula 2 [5,6,7].

Coumarin is a lactone widely present in plants. It has various biological activities, such as anticancer, antioxidant, antiallergic, and anti-inflammatory activities [8,9,10]. Dicoumarin is a coumarin derivative with a variety of biological activities, including anti-inflammatory, antibacterial, antitumor, anti-HCV, anti-HIV, antidementia, antimalaria, and antifungal activities [11]. There have been no reports on the use of dicoumarin as an antiallergic drug. Therefore, in the current study, an effective method for synthesizing dicoumarin derivatives was developed, and the antiallergic activities of the synthesized dicoumarin derivatives were studied. There have been several methods for synthesizing dicoumarin derivatives, including the use of Lewis acid catalysts [12,13], phase transfer catalysts [14,15], alkaline catalysts [16,17], or microwave-assisted synthesis [18,19]. However, most of these methods are not suitable for industrial production because they can cause environmental pollution, they require expensive catalysts, or their product yields are low.

In this study, 14 dicoumarin derivatives were synthesized by an easy-to-scale-up method whereby diethylamine was used as both a base and a catalyst, and some of them had good inhibitory effects on the degranulation of rat basophilic leukemia cells (RBL-2H3 cells) [20] and mouse bone-marrow-derived mast cells (mBMMCs) [21]. This study provides an idea for synthesizing new antiallergic drugs.

## 2. Results and Discussion

### 2.1. Synthesis of Dicoumarin Derivatives

#### 2.1.1. Selection of the Optimal Catalyst

Table 1 shows the parameters of an experiment for determining the optimal catalyst for the production of compound **3a** through the condensation reaction between 4-hydroxycoumarin and paraformaldehyde. When anhydrous ethanol and sulfuric acid were used as the reaction solvent and the catalyst, respectively (entry 1), the product yield was very low (6%), showing that the reaction was slow under acidic conditions. Therefore, the reaction was carried out under basic conditions. When triethylamine was used as the catalyst, the product yield was 25% after 12 h of reaction (entry 2), which is similar to that obtained by Khan K M et al. [17]. When pyrrolidine was used as the catalyst, the substrates were completely consumed in 3 h, and the product yield was 60% (entry 3). When diethylamine, which is both nucleophilic and basic, was used as the catalyst at a dosage of 1 equivalent [22], the substrates were completely consumed in 3 h, and the product yield was 87% (entry 4). As the dosage of diethylamine increased to 2 equivalents, the product yield increased to 95% (entry 5). However, a further increase in the diethylamine dosage to 3 equivalents reduced the product yield to 90% (entry 6). On the basis of the above results, diethylamine at a dosage of 2 equivalents was selected as the optimal catalyst for the production of compound **3a**.

#### 2.1.2. Selection of the Optimal Solvent

The optimal solvent for the production of compound **3a** (the compound produced through the condensation reaction between 4-hydroxycoumarin and paraformaldehyde) was selected by using diethylamine at a dose of 2 equivalents as the catalyst. Of the different tested solvents (Table 2), anhydrous ethanol exhibited the highest product yield (95%). Therefore, it was selected as the optimal solvent for the production of compound **3a**.

#### 2.1.3. Selection of the Optimal Reaction Temperature

The optimal reaction temperature for the production of compound **3a** was selected by using diethylamine at a dose of 2 equivalents as the catalyst and anhydrous ethanol as the solvent (Table 3). A 9.9% product yield was obtained at room temperature, and a 57% product yield was obtained at 50 °C, indicating the significant impact of temperature on the product yield. However, the product yield obtained at 90 °C (entry 4) was not significantly different from that obtained at 80 °C (entry 3). On the basis of the above results, 80 °C was selected as the optimal reaction temperature for the production of compound **3a**.

#### 2.1.4. Production of Dicoumarin Derivatives from Aldehyde Substrates Other than Paraformaldehyde

On the basis of the optimal reaction conditions for the synthesis of compound **3a**, compounds **3b**–**3n** were synthesized from aldehyde substrates other than paraformaldehyde (Table 4). The product yield obtained when benzaldehyde (entry 2) or 2-bromo-5-hydroxybenzaldehyde (entry 3) was used as the aldehyde substrate was 95% or 93%, respectively. When isobutyraldehyde was used as the aldehyde substrate, the product yield was 95% (entry 4). When 3-hydroxybenzaldehyde (entry 5) or furfural (entry 6) was used as the aldehyde substrate, the product yield was 87% or 91%, respectively. The product yield obtained when 3-phenylpropanal (entry 7) or pyridine-4-carbaldehyde (entry 8) was used as the aldehyde substrate was 86% or 73%, respectively. When 3-methylthiophene-2-carbaldehyde (entry 9) or 3,4-dimethoxybenzaldehyde (entry 10) was used as the aldehyde substrate, the product yield was 88% or 85%, respectively. The product yield obtained using methoxybenzaldehyde was 93% (entry 11), while that obtained using 4-hydroxy-3-methoxybenzaldehyde was 72% (entry 12). The product yield obtained using 4-hydroxybenzaldehyde was 45% (entry 13), and that obtained using tetrametric (metaldehyde) acetaldehyde (entry 14) was 87%. In order to show the merit of our newly developed procedures, we compared our results for the synthesis of 3,3′-(phenylmethylene)bis(4-hydroxy-2H-chromen-2-one) using the Diethylamine with the results of some of the catalysts reported in the literature for the same transformation (Table 5). The procedure has several advantages such as high reaction rates, ease of preparation and handling of the catalyst, simple and green experimental procedure, and use of an inexpensive catalyst.

### 2.2. Antiallergic Activity of Dicoumarin Derivatives

#### 2.2.1. Inhibitory Effects of Dicoumarin Derivatives on the Degranulation of RBL-2H3 Cells

**Figure 1:**
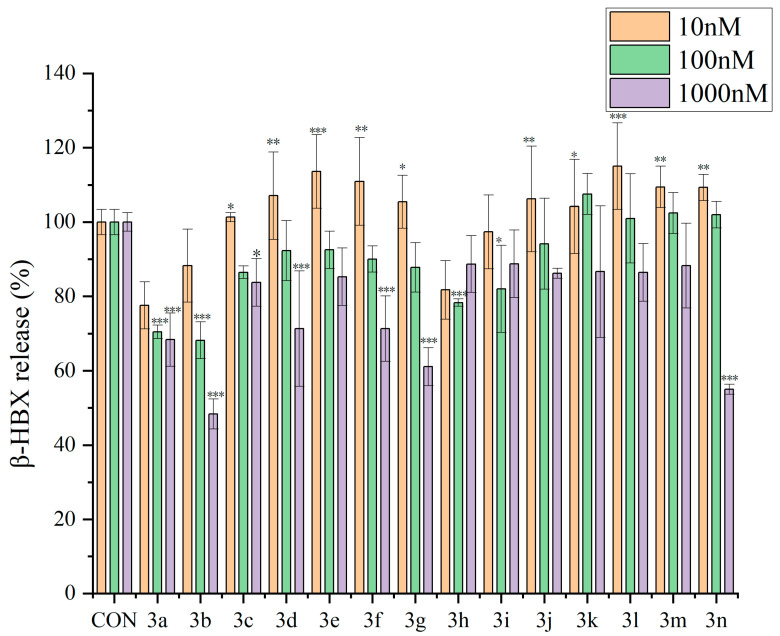
Inhibitory effects of compounds **3a**–**3n** on the release of β-HEX from RBL-2H3 cells. Data are expressed as means ± standard deviations (*n* = 3). * *p* < 0.05, ** *p* < 0.01, and *** *p* < 0.001 vs. the control (CON) group.

RBL-2H3 cells were used for an in vitro anaphylaxis assay, and the degranulation of the cells was detected by using the release of β-hexosaminidase (β-HEX) from the cells as a marker. All of the compounds **3a**–**3n** inhibited the release of β-HEX from RBL-2H3 cells, with compound **3b** showing a 48.38% inhibition rate when its concentration was 1 µM (Figure 1). Moreover, the inhibition rates of compounds **3a**, **3b**, **3d**, **3f**, **3g**, and **3n** were generally higher than those of the other eight compounds, especially at a 1 µM concentration. At a concentration of 10 nM, compounds **3a**, **3b**, **3h**, and **3i** showed inhibitory effects on the release of β-HEX from RBL-2H3 cells, with compound **3a** demonstrating the most significant inhibitory effect. The inhibition rate of compound **3a** increased with an increase in the concentration of the compound, reaching 31.62% when the compound concentration was 1 µM. Except for compounds **3h**, **3i**, and **3k**, compounds **3a**–**3n** exhibited enhancements in their inhibitory effects on the release of β-HEX from RBL-2H3 cells when their concentrations increased. The above results demonstrated the antiallergic activity of compounds **3a**–**3n**.

**Figure 2:**
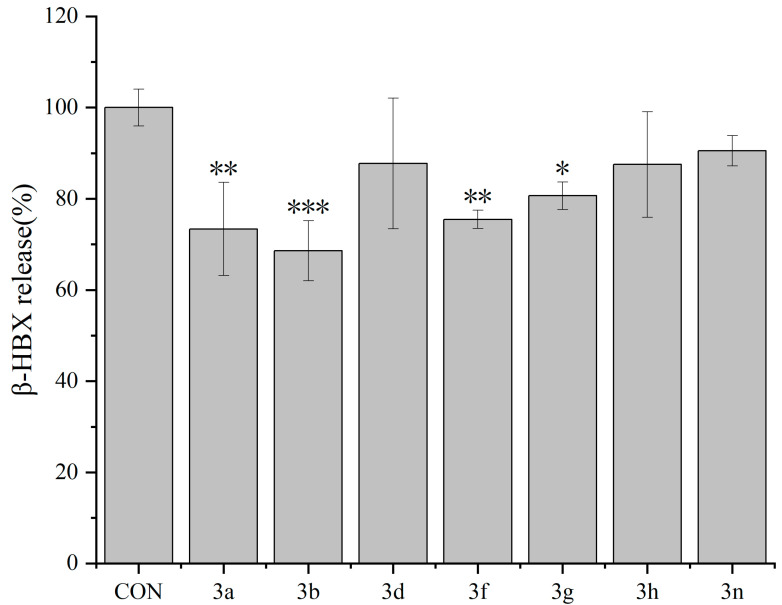
Inhibitory effects of compounds **3a**, **3b**, **3d**, **3f**, **3g**, and **3n** (500 nM each) on the release of β-HEX from RBL-2H3 cells. Data are expressed as means ± standard deviations (*n* = 3). * *p* < 0.05, ** *p* < 0.01, and *** *p* < 0.001 vs. the control (CON) group.

At a concentration of 500 nM, compounds **3a**, **3b**, **3d**, **3f**, **3g**, and **3n** showed inhibitory effects on the release of β-HEX from RBL-2H3 cells, with compound **3b** showing the best inhibitory activity (Figure 2).

#### 2.2.2. Inhibitory Effects of Dicoumarin Derivatives on the Degranulation of mBMMCs

**Figure 3:**
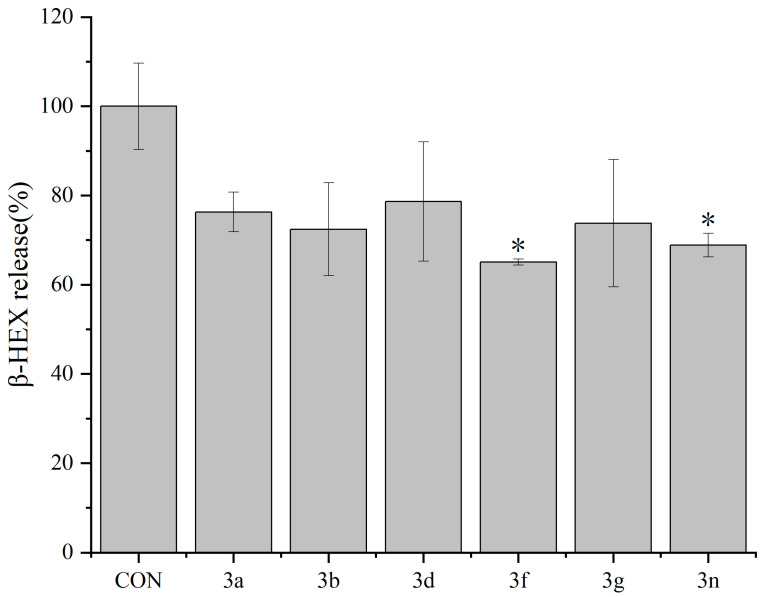
Inhibitory effects of compounds **3a**, **3b**, **3d**, **3f**, **3g**, and **3n** (1 µM each) on the release of β-HEX from mBMMCs. Data are expressed as means ± standard deviations (*n* = 3). * *p* < 0.05 vs. the control (Con) group.

Furthermore, we examined the effects of compounds on the other allergy cell model, mouse bone-marrow-derived mast cells. The inhibitory effects of compounds **3a**, **3b**, **3d**, **3f**, **3g**, and **3n** (the top six compounds in the study in terms of their inhibitory effects of compounds **3a**–**3n** on the release of β-HEX from RBL-2H3 cells) on the release of β-HEX from mBMMCs were evaluated (Figure 3). The concentration of each compound was 1 µM. All of the compounds inhibited the release of β-HEX from mBMMCs, with compound **3f** showing the best inhibitory activity, suggesting that dicoumarin derivative can inhibit the basophilic leukemia cells and mast cells.

## 3. Materials and Methods

### 3.1. General Experimental Procedures

All chemical reagents were obtained from commercial suppliers (Aladdin, Macklin, Energy Chemical, Sinopharm Chemical Reagent Co., Ltd., Shanghai, China) and were used without further purification. Proton nuclear magnetic resonance (^1^H NMR) spectra were recorded on Bruker 400 (400 MHz) NMR spectrometers and a Bruker AVANCE NEO 600 (600 MHz) at 23 °C. Proton chemical shifts are expressed in parts per million (ppm, δ scale) relative to residual protium in the NMR solvent (CHCl_3_, δ 7.26, and DMSO-d6, δ 2.50). Data are presented as follows: chemical shift, multiplicity (s = singlet, d = doublet, t = triplet, q = quartet, like-t = like-triplet, like-td = like-triple doublet, m = multiplet, and/or multiple resonances), coupling constant (*J*) in Hertz (Hz), and integration. Carbon nuclear magnetic resonance (^13^C NMR) spectra were recorded on Bruker 400 (100 MHz) NMR spectrometers and Bruker AVANCE NEO 600 (150 MHz) at 23 °C. Carbon chemical shifts are expressed in parts per million (ppm, δ scale) relative to the carbon resonances of the NMR solvent (CDCl_3_, δ 77.16, and DMSO-d6, δ 39.60). Infrared spectra were obtained using an IRAffinity-1s infrared spectrometer. Data are presented as follows: frequency of absorption (cm^−1^) and intensity of absorption. Mass spectra were recorded on a 6540 UHD Accurate-Mass Q-TOF mass spectrometer (Agilent Technologies, Santa Clara, CA, USA). The purity of all samples was recorded on an Agilent 1200 high-performance liquid chromatograph. The purity of all samples was above 90%. An Agilent 1200 series HPLC with a Discovery C_18_ (4.6 × 250 mm, 5μm particle sizes) reversed-phase column was used for analytical HPLC analyses. The elution buffer was an A/B gradient, where A = 0.1% CF_3_COOH in H_2_O and B = CH_3_CN (gradient elution: 0–20 min, 5%B-95%B). Melting points were recorded using an X-4 micro melting point apparatus and were uncorrected.

### 3.2. Synthesis and Characterization of the Compounds

#### 3.2.1. General Approach to the Preparation of Compounds **3a**–**3n**

4-Hydroxycoumarin (5 g, 0.03 mol) was placed in a 250 mL round-bottom flask and dissolved in 30 mL of anhydrous ethanol, followed by the addition of an aldehyde (0.015 mol) and then diethylamine (3.16 mL, 0.03 mol) dropwise. The mixture was heated to reflux and stirred for 3–5 h. The reaction was monitored via TLC until the disappearance of the starting material and the appearance of a single product spot. The pH was adjusted to 1–2 with 10% dilute hydrochloric acid, and 20 mL of cold water was added. The mixture was stirred at room temperature for 20 min, and some solids were precipitated. The reaction mixture was filtered under reduced pressure, and the filter cake was washed with 10 mL of cold water and dried under vacuum to obtain a white solid crude product. The white crude product was then added to a 250 mL round-bottom flask, followed by the addition of 100 mL of cold water, and the mixture was stirred for 4 h at room temperature. The mixture was filtered under reduced pressure and rinsed with 20 mL of cold water, and the filter cake was dried to obtain the final product.

#### 3.2.2. 3,3′-Methylenebis(4-hydroxy-2H-chromen-2-one) (**3a**)

White solid, yield 95%; m.p. 260–262 °C, (lit.: 264–265 °C), the ^1^H and ^13^C NMR spectral data correspond to the literature data [23]; IR (KBr, cm^−1^): 3432, 1695, 1521, 1273, 1159, 1058, 833, 763, 680; HRMS-ESI (*m*/*z*): [M + H]^+^ calcd for C_19_H_13_O_6_^+^ 337.0712, found: 337.0708.

#### 3.2.3. 3,3′-(Phenylmethylene)bis(4-hydroxy-2H-chromen-2-one) (**3b**)

White solid, yield 95%; m.p. 143–145 °C, (lit.: 231–232 °C), the ^1^H and ^13^C NMR spectral data correspond to the literature data [24]; IR (KBr, cm^−1^): 3021, 1662, 1616, 1182, 1107, 797, 759, 696; HRMS-ESI (*m*/*z*): [M + H]^+^ calcd for C_25_H_17_O_6_^+^ 413.1020, found: 413.1022.

Possible polymerization of phencyclic bicoumarins with diethylamine to form ammonium salts.

#### 3.2.4. 3,3′-((2-Bromo-5-hydroxyphenyl)methylene)bis(4-hydroxy-2H-chromen-2-one) (**3c**)

White solid, yield 93%; m.p. 160–161 °C; ^1^H NMR(400 MHz, DMSO-*d*6): δ 9.30 (s, 1H), 8.17 (s, 2 H), 7.82 (dd, *J* = 7.72, 1.32 Hz, 2 H), 7.51~7.47 (m, 2 H), 7.26~7.21 (m, 4 H), 7.16 (d, *J* = 8.48 Hz, 1 H), 6.88 (d, *J* = 2.48 Hz, 1 H), 6.46 (dd, *J* = 8.48, 2.88 Hz, 1H), 5.91 (s, 1 H), 2.92 (q, *J* = 6.44 Hz, 4H), 1.16 (t, *J* = 7.24 Hz, 6H); ^13^C NMR (100 MHz, DMSO-d6): δ 168.06, 164.36, 156.50, 152.88, 143.35, 133.58, 131.22, 124.52, 123.33, 120.46, 118.46, 115.90, 114.88, 112.29, 103.36, 41.87, 38.81, 11.50; IR (KBr, cm^−1^): 3417, 1653, 1616, 1558, 1107, 808, 758, 677; HRMS-ESI (*m*/*z*): [M + H]^+^ calcd for C_25_H_16_BrO_7_^+^ 507.0074, found: 507.0077.

#### 3.2.5. 3,3′-(2-Methylpropane-1,1-diyl)bis(4-hydroxy-2H-chromen-2-one) (**3d**)

White solid, yield 95%; m.p. 196–198 °C, (lit.: 199–200 °C) [25];^1^H NMR (400 MHz, DMSO-*d*6): δ 7.95 (d, *J* = 7.76 Hz, 2 H), 7.60 (t, *J* = 7.32 Hz, 2 H), 7.35 (d, *J* = 7.48 Hz, 4 H), 4.55 (d, *J* = 11.60 Hz, 1 H), 3.08–3.00 (m, 1 H), 0.87 (d, *J* = 6.44 Hz, 6 H); ^13^C NMR (100 MHz, DMSO-*d*6): δ 165.58, 164.70, 152.42, 132.21, 124.23, 116.32, 112.99, 105.30, 26.18, 21.78; IR (KBr, cm^−1^): 3132, 2964, 1681, 1608, 1558, 1456, 1394, 1276, 1031, 763; HRMS-ESI (*m*/*z*): [M + H]^+^ calcd for C_22_H_19_O_6_^+^ 379.1176, found: 379.1178.

#### 3.2.6. 3,3′-((3-Hydroxyphenyl)methylene)bis(4-hydroxy-2H-chromen-2-one) (**3e**)

White solid, yield 87%; m.p. 198–200 °C, (lit.: 210.5 °C), the ^1^H and ^13^C NMR spectral data correspond to the literature data [17]; IR (KBr, cm^−1^): 3082, 1662, 1616, 1566, 1348, 1101, 910, 810, 761, 688; HRMS-ESI (*m*/*z*): [M + H]^+^ calcd for C_25_H_17_O_7_^+^ 429.0969, found: 429.0971.

#### 3.2.7. 3,3′-(Furan-2-ylmethylene)bis(4-hydroxy-2H-chromen-2-one) (**3f**)

Yellow solid, yield 91%; m.p. 220–222 °C, (lit.: 194–196 °C), the ^1^H and ^13^C NMR spectral data correspond to the literature data [26]; IR (KBr, cm^−1^): 3080, 1654, 1618, 1560, 1350, 1097, 765; HRMS-ESI (*m*/*z*): [M + H]^+^ calcd for C_23_H_15_O_7_^+^ 403.0813, found: 403.0810.

#### 3.2.8. 3,3′-(3-Phenylpropane-1,1-diyl)bis(4-hydroxy-2H-chromen-2-one) (**3g**)

Yellow solid, yield 86%; m.p. 195–197 °C, (lit.: 197–198 °C) [27]; ^1^H NMR (400 MHz, DMSO-*d*6): δ 7.96~7.93 (m, 2 H), 7.60~7.56 (m, 2 H), 7.35~7.31 (m, 4 H), 7.23~7.20 (m, 2 H), 7.16~7.10 (m, 3 H), 4.94 (t, *J* = 8.08 Hz, 1 H), 2.55~2.50 (m, 2 H), 2.45~2.40 (m, 2 H); ^13^C NMR (100 MHz, DMSO-*d*6): δ 165.15, 165.05, 152.48, 142.58, 132.12, 128.96, 128.91, 128.64, 128.61, 125.99, 124.42, 124.15, 118.32, 116.32, 105.52, 34.59, 32.42, 32.22; IR (KBr, cm^−1^): 1654, 1618, 1604, 1456, 1325, 1273, 1176, 1101, 769, 756; HRMS-ESI (*m*/*z*): [M + H]^+^ calcd for C_27_H_21_O_6_^+^ 441.1333, found: 441.1334.

#### 3.2.9. 3,3′-(Pyridin-4-ylmethylene)bis(4-hydroxy-2H-chromen-2-one) (**3h**)

White solid, yield 73%; m.p. 231–234 °C, (lit.: 218 °C), the ^1^H and ^13^C NMR spectral data correspond to the literature data [28]; IR (KBr, cm^−1^): 3080, 1683, 1610, 1496, 1205, 1180, 1107, 761; HRMS-ESI (*m*/*z*): [M + H]^+^ calcd for C_24_H_16_NO_6_^+^ 414.0972, found: 414.0973.

#### 3.2.10. 3,3′-((3-Methylthiophen-2-yl)methylene)bis(4-hydroxy-2H-chromen-2-one) (**3i**)

Deep green solid, yield 88%; m.p. 130–132 °C, (lit.: 134–136 °C), the ^1^H and ^13^C NMR spectral data correspond to the literature data [26]; IR (KBr, cm^−1^): 2995, 2495, 1672, 1606, 1558, 1180, 1107, 1029, 760; HRMS-ESI (*m*/*z*): [M + H]^+^ calcd for C_27_H_17_O_6_S^+^ 433.0741, found: 433.0743.

#### 3.2.11. 3,3′-((3,4-Dimethoxyphenyl)methylene)bis(4-hydroxy-2H-chromen-2-one) (**3j**)

White Solid, yield 85%; m.p. 266–268 °C, (lit.: 262–264 °C), the ^1^H and ^13^C NMR spectral data correspond to the literature data [29]; IR (KBr, cm^−1^): 3647, 2935, 2607, 1660, 1614, 1516, 1267, 1139, 1097, 1028, 765; HRMS-ESI (*m*/*z*): [M + H]^+^ calcd for C_27_H_21_O_8_^+^ 473.1231, found: 473.1233.

#### 3.2.12. 3,3′-((4-Methoxyphenyl)methylene)bis(4-hydroxy-2H-chromen-2-one) (**3k**)

Yellowish white solid, yield 93%; m.p. 243–244 °C, (lit.: 240–242 °C), the ^1^H and ^13^C NMR spectral data correspond to the literature data [29]; IR (KBr, cm^−1^): 3649, 3007, 2837, 2723, 2607, 1666, 1564, 1311, 1255, 1093, 767; HRMS-ESI (*m*/*z*): [M + H]^+^ calcd for C_26_H_19_O_7_^+^ 443.1126, found: 443.1127.

#### 3.2.13. 3,3′-((4-Hydroxy-3-methoxyphenyl)methylene)bis(4-hydroxy-2H-chromen-2-one) (**3l**)

White solid, yield 72%; m.p. 249–251 °C, (lit.: 254–259 °C), the ^1^H NMR spectral data correspond to the literature data [30]; ^13^C NMR (100 MHz, DMSO-*d*6): δ 165.64, 165.18, 152.66, 147.80, 145.14, 132.21, 131.09, 124.32, 124.13, 119.68, 118.52, 116.37, 115.59, 112.21, 104.92, 56.27, 36.06; IR (KBr, cm^−1^): 3500, 3088, 2731, 2596, 1670, 1614, 1350, 1271, 1207, 1093, 765; HRMS-ESI (*m*/*z*): [M + H]^+^ calcd for C_26_H_19_O_8_^+^ 459.1075, found: 459.1075.

#### 3.2.14. 3,3′-((4-Hydroxyphenyl)methylene)bis(4-hydroxy-2H-chromen-2-one) (**3m**)

Yellow solid, yield 81%; m.p. 220–223 °C, (lit.: 220–222 °C), the ^1^H and ^13^C NMR spectral data correspond to the literature data [23]; IR (KBr, cm^−1^): 3080, 2727, 2605, 1668, 1618, 1560, 1508, 1350, 1105, 906, 759; HRMS-ESI (*m*/*z*): [M + H]^+^ calcd for C_25_H_17_O_7_^+^ 429.0969, found: 429.0969.

#### 3.2.15. 3,3′-(Ethane-1,1-diyl)bis(4-hydroxy-2H-chromen-2-one) (**3n**)

Yellow solid, yield 87%; m.p. 173–175 °C, (lit.: 176–178 °C), the ^13^C NMR spectral data correspond to the literature data [25]; ^1^H NMR (600 MHz, DMSO-*d*6): δ 7.86 (dd, *J* = 7.80, 1.62 Hz, 2 H), 7.47~7.44 (m, 2 H), 7.23~7.18 (m, 4 H), 4.98 (q, *J* = 7.62 Hz, 1 H), 1.49 (d, *J* = 7.68 Hz, 3 H); IR (KBr, cm^−1^): 2968, 2927, 1664, 1610, 1541, 1417, 1373, 1195, 1112, 1012, 761; HRMS-ESI (*m*/*z*): [M + H]^+^ calcd for C_20_H_15_O_6_^+^ 351.0863, found: 351.0863.

### 3.3. Compounds Anti-Allergy Activity Assay

#### 3.3.1. RBL-2H3 Cell Culture

Rat basophilic leukemia cells (RBL-2H3) were purchased from Pericella Life Science and Technology Co., Ltd. (Wuhan, China). The cells were cultured in DMEM medium supplemented with 10% heat-inactivated fetal bovine serum, 100 U/mL penicillin, and 100 μg/mL streptomycin at 37 °C in a 5% CO_2_ incubator.

#### 3.3.2. Isolation, Culture, and Identification of Mouse Bone Marrow-Derived Mucosal Mast Cells

Male BALB/c mice (6 weeks old) were sacrificed and cleaned with 70% alcohol, and the entire hind limb was clipped along the hip joint. The skin was then removed on an ultra-clean table, and the tibia and femur were extracted. A 1 mL syringe needle was inserted into the bone marrow cavity to flush out the bone marrow. Erythrocytes were removed, and the bone marrow cell precipitate was obtained through centrifugation, with the supernatant discarded. The cells were cultured in an RPMI 1640 medium with 10% heat-inactivated fetal bovine serum, 20 mM HEPES, 1 mM sodium pyruvate, 100 µM MEM, 10 µM 2-mercaptoethanol, 100 U/mL PS, and 2 µg/mL gentamicin solution, 20 ng/mL recombinant mouse IL-3, 40 ng/mL recombinant mouse SCF, 5 ng/mL recombinant mouse IL-9, and 1 ng/mL TGF-β1, and maintained in an incubator at 37 °C with 5% CO_2_. The culture medium was changed every four days. After four weeks, the expression of cell surface markers FcεRI and CD117 was detected through flow cytometry, and the cells were considered suitable for experiments when the double-positive cells exceeded 95%.

#### 3.3.3. β-Hexosaminidase Release Experiment in RBL-2H3 Cells

The extent of degranulation was assessed through the measurement of the secretion of β-Hex. RBL-2H3 cells were suspended at a density of 1 × 10^5^ cells/mL and sensitized with 0.5 μg/mL DNP-IgE at 37 °C for 12 h. The sensitized cells were then washed with Tyrode’s buffer (130 mM NaCl, 5 mM KCl,1.4 mM CaCl_2_, 1 mM MgCl_2_, 5.6 mM glucose, 10 mM HEPES, and 0.1% BSA, pH 7.4), and the cell pellet was incubated for 30 min at 37 °C, with the addition of 0 nM, 10 nM, 100 nM, 500 nM, and 1 μM bis-coumarin compounds, followed by stimulation with 100 ng/mL DNP-BSA for 1 h. The cell supernatant was centrifuged and collected, and the cell pellet was lysed with Tyrode’s buffer containing 0.5% Triton X-100. After incubation with the substrate at 37 °C for 1 h, the reaction was terminated through the addition of 0.2 M sodium hydroxide and 0.2 M glycine. The OD values of the supernatant and cell lysates at 405 nm were determined using an enzyme marker. The percentage of β-hexosaminidase release was calculated using the formula: β-hexosaminidase (%) = (supernatant OD)/(supernatant OD + cell OD) ×100%.

#### 3.3.4. β-Hexosaminidase Release Experiment in mBMMCs

The extent of degranulation was assessed through the measurement of the secretion of β-Hex. mBMMCs were suspended at a density of 2 × 10^5^ cells/mL and sensitized with 1.5 μg/mL DNP-IgE for 6 h at 37 °C. The sensitized cells were washed with Tyrode’s buffer, and 1 μM of bicoumarin analogs were added separately, followed by incubation for 30 min at 37 °C. The mBMMCs were then stimulated with 100 ng/mL DNP-BSA for 1 h. The cell supernatant was centrifuged and collected, and the cell pellet was lysed with Tyrode’s buffer containing 0.5% Triton X-100. After incubation with the substrate at 37 °C for 1 h, the reaction was terminated through the addition of 0.2 M sodium hydroxide and 0.2 M glycine. The OD values of the supernatant and cell lysates at 405 nm were determined using an enzyme marker. The percentage of β-hexosaminidase release was calculated using the formula: β-hexosaminidase (%) = (supernatant OD)/(supernatant OD + cell OD) × 100%.

## 4. Conclusions

In this study, an effective method for synthesizing dicoumarin derivatives was developed on the basis of a diethylamine-catalyzed one-pot Knoevenagel–Michael reaction; 14 dicoumarin derivatives were synthesized using the method, and a mechanism for the formation of dicoumarin derivatives through the one-pot reaction was proposed. The post-processing of the dicoumarin derivatives did not require chromatographic separation, and the purification of the compounds was carried out by pulping and recrystallization, making the method conducive to industrial production. After the 14 dicoumarin derivatives were obtained, their antiallergic activities were tested.

The inhibitory effects of the 14 dicoumarin derivatives on the release of β-HEX from RBL-2H3 cells and mBMMCs (the two types of mast cells commonly used in research on allergies) were tested. Among the 14 compounds, compound **3b**, which was a dicoumarin derivative obtained by using benzaldehyde as its aldehyde substrate, exhibited the strongest inhibitory effect on the release of β-HEX from RBL-2H3 cells, while compound **3g**, which was a dicoumarin derivative obtained by using 3-phenylpropanal as its aldehyde substrate, exhibited the strongest inhibitory effect on the release of β-HEX from mBMMCs. This study demonstrates that dicoumarin derivatives can effectively inhibit the degranulation of mast cells and suppress IgE-mediated allergic reactions, providing new antiallergic drugs.

## Figures and Tables

**Table 1:** Selection of the optimal catalyst for the production of compound **3a**.

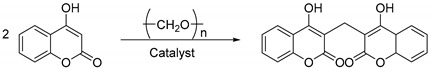				
Entry	Catalyst	Catalyst Dosage/eq.	Time/h	Yield/%
1	H_2_SO_4_	2	12	6
2	Triethylamine	2	12	25
3	Pyrrolidine	2	3	60
4	Diethylamine	1	3	87
5	Diethylamine	2	3	95
6	Diethylamine	3	3	90

Reaction conditions: 4-hydroxycoumarin (5 g, 0.03 mol), paraformaldehyde (0.46 g, 0.015 mol), and anhydrous ethanol (30 mL). The reaction system was protected with nitrogen gas, and the reaction time was determined on the basis of the detection of a single spot on the TLC plate.

**Table 2:** Selection of the optimal solvent for the production of compound **3a**.

Entry	Solvent	Yield/%
1	Anhydrous ethanol	95
2	95% ethanol	80
3	MeOH	80
4	CH_3_CN	45
5	CH_2_Cl_2_	50
6	DMSO	75

Reaction conditions: 4-hydroxycoumarin (5 g, 0.03 mol), paraformaldehyde (0.46 g, 0.015 mol), and diethylamine (2 equivalents (3.16 mL, 0.03 mol)). The reaction system was protected with nitrogen gas, and the reaction time was 3 h.

**Table 3:** Selection of the optimal reaction temperature for the production of compound **3a**.

Entry	Temperature/°C	Yield/%
1	room temperature	9.9
2	50	57
3	80	95
4	90	95

Reaction conditions: 4-hydroxycoumarin (5 g, 0.03 mol), paraformaldehyde (0.46 g, 0.015 mol), diethylamine (2 equivalents (3.16 mL, 0.03 mol)), and ethanol (30 mL). The reaction system was protected with nitrogen gas, and the reaction time was 3 h.

**Table 4:** Aldehyde substrates, reaction time, and yields of compounds **3a**–**3n**.

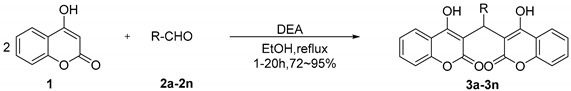						
R = -H, -C_6_H_5_, -C_6_H_4_BrO, -C_3_H_7_, -C_6_H_5_O, -C_4_H_3_O, -C_8_H_9_, -C_5_H_4_N, -C_5_H_5_S, -C_8_H_9_O_2_, -C_7_H_7_O, -C_7_H_7_O_2_, -C_6_H_5_O, or -CH_3_.						
Entry	Aldehyde	Product	Time/h	Yield/%	M.p. (°C)	
					Obs.	Lit.
1	HCHO	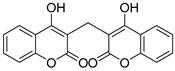 **3a**	3	95	260–262	264–265 [23]
2	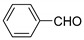	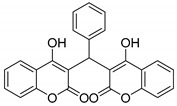 **3b**	4	95	143–145	231–232 [24]
3	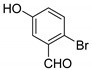	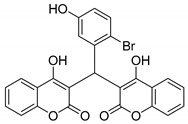 **3c**	5	93	160–161	-
4	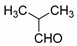	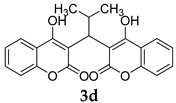 **3d**	5	95	196–198	199–200 [25]
5	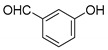	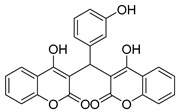 **3e**	3.5	87	198–200	210.5 [17]
6	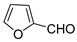	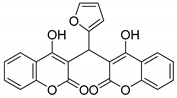 **3f**	4	91	220–222	194–196 [26]
7	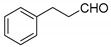	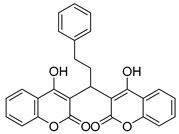 **3g**	1	86	195–197	197–198 [27]
8	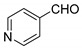	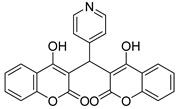 **3h**	2	73	231–234	218 [28]
9	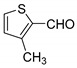	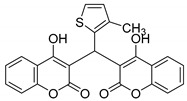 **3i**	3	88	130–132	134–136 [26]
10	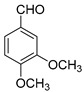	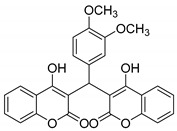 **3j**	4	85	266–268	262–264 [29]
11	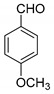	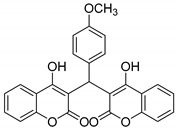 **3k**	7	93	243–244	240–242 [29]
12	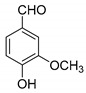	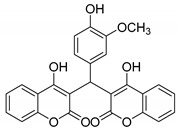 **3l**	5	72	249–251	254–259 [30]
13	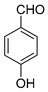	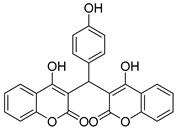 **3m**	20	81	220–223	220–222 [23]
14	CH_3_CHO	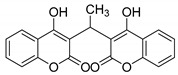 **3n**	5.5	87	173–175	176–178 [25]

Reaction conditions: 4-hydroxycoumarin (5 g, 0.03 mol), an aldehyde (0.015 mol), diethylamine (2 equivalents (3.16 mL, 0.03 mol)), and anhydrous ethanol (30 mL). The reaction system was protected with nitrogen gas.

**Table 5:** Comparison results of Diethylamine with other catalysts reported in the literature using **5a** as a model compound.

Entry	Catalyst	Reaction Conditions	Time (h)	Yield/(%)	References
1	SDS (20 mol%)	H_2_O, 60 °C	2.3	90	[17]
2	[P_4_VPy-BuSO_3_H]HSO_4_	Toluene, 90 °C	0.8	93	[31]
3	Lipase (PPL)	EtOH, 45 °C	6	85	[32]
4	Et_3_N	EtOH, RT	24	90	[23]
5	piperidine	EtOH, RT	4	92	[17]
7	Diethylamine (2 eq.)	EtOH, reflux	3	95	This work

## Data Availability

Data are contained within the article and the Supplementary Materials.

## Supplementary Materials

The following supporting information can be downloaded at: https://www.mdpi.com/article/10.3390/molecules29163799/s1, ^1^H NMR and ^13^C NMR spectra for the synthesized compounds and ESI-MS spectra.
