# Supplementary material for: Synthesis and Antiallergic Activity of Dicoumarin Derivatives
Source: Molecules. 2024 Aug 10;29(16):3799. doi: 10.3390/molecules29163799 (PMC11357222; doi:10.3390/molecules29163799)
Supplement: Supplementary file 1 [file molecules-29-03799-s001.zip › molecules-3113277-supplementary.pdf]

Supporting Information For

Research on the synthesis of dicoumarol derivatives and antiallergic activity

Zhang Yuying [1], Wang Xiaoyu [2*], Zhou Dejun [1*]

[1] The key laboratory of Chinese medicine research and development in HeBei province, Traditional Chinese Medicine Institute of Chengde Medical University,Chengde, 067000, China; 2539963506@qq.com (Z.Y.); zhoudj20220307@163.com (Z.D.);

[2] Experiment Center for Science and Technology, Shanghai University of Traditional Chinese Medicine, 1200 Cailun Road, Shanghai 201203, China;

* Correspondence: zhoudj20220307@163.com (Z.D.); 0000002733@shutcm.edu.cn (W.X.);

CATALOG

Figure S 1 ^{1}H NMR spectra of compound 3a........1

Figure S 2 ^{13}C NMR spectra of compound 3a........1

Figure S 3 MS spectra of compound 3a........2

Figure S 4 The purity of 3a from HPLC........3

Figure S 5 The purity of 3a from FTIR........3

Figure S 6 ^{1}H NMR spectra of compound 3b........4

Figure S 7 ^{13}C NMR spectra of compound 3b........4

Figure S 8 MS spectra of compound 3b........5

Figure S 9 The purity of 3b from HPLC........6

Figure S 10 The purity of 3b from FTIR........6

Figure S 11 ^{1}H NMR spectra of compound 3c........7

Figure S 12 ^{13}C NMR spectra of compound 3c........7

Figure S 13 MS spectra of compound 3c........8

Figure S 14 The purity of 3c from HPLC........9

Figure S 15 The purity of 3c from FTIR........9

Figure S 16 ^{1}H NMR spectra of compound 3d........10

Figure S 17 ^{13}C NMR spectra of compound 3d........10

Figure S 18 MS spectra of compound 3d........11

Figure S 19 The purity of 3d from HPLC........12

Figure S 20 The purity of 3d from FTIR........12

Figure S 21 ^{1}H NMR spectra of compound 3e........13

Figure S 22 ^{13}C NMR spectra of compound 3e........13

Figure S 23 MS spectra of compound 3e 14
Figure S 24 The purity of 3e from HPLC 15
Figure S 25 The purity of 3e from FTIR 15
Figure S 26 ^{1}H NMR spectra of compound 3f 16
Figure S 27 ^{13}C NMR spectra of compound 3f 17
Figure S 28 MS spectra of compound 3f 18
Figure S 29 The purity of 3f from HPLC 19
Figure S 30 The purity of 3f from FTIR 19
Figure S 31 ^{1}H NMR spectra of compound 3g 20
Figure S 32 ^{13}C NMR spectra of compound 3g 20
Figure S 33 MS spectra of compound 3g 21
Figure S 34 The purity of 3g from HPLC 22
Figure S 35 The purity of 3g from FTIR 22
Figure S 36 ^{1}H NMR spectra of compound 3h 23
Figure S 37 ^{13}C NMR spectra of compound 3h 24
Figure S 38 MS spectra of compound 3h 25
Figure S 39 The purity of 3h from HPLC 26
Figure S 40 The purity of 3h from FTIR 26
Figure S 41 ^{1}H NMR spectra of compound 3i 27
Figure S 42 ^{13}C NMR spectra of compound 3i 27
Figure S 43 MS spectra of compound 3i 28
Figure S 44 The purity of 3i from HPLC 29
Figure S 45 The purity of 3i from FTIR 29
Figure S 46 ^{1}H NMR spectra of compound 3j 30
Figure S 47 ^{13}C NMR spectra of compound 3j 31
Figure S 48 MS spectra of compound 3j 32
Figure S 49 The purity of 3j from HPLC 33
Figure S 50 The purity of 3j from FTIR 33
Figure S 51 ^{1}H NMR spectra of compound 3k 34
Figure S 52 ^{13}C NMR spectra of compound 3k 34
Figure S 53 MS spectra of compound 3k 35
Figure S 54 The purity of 3k from HPLC 36
Figure S 55 The purity of 3k from FTIR 36

Figure S 56 ^{1}H NMR spectra of compound 3l 37
Figure S 57 ^{13}C NMR spectra of compound 3l 38
Figure S 58 MS spectra of compound 3l 39
Figure S 59 The purity of 3l from HPLC 40
Figure S 60 The purity of 3l from FTIR 40
Figure S 61 ^{1}H NMR spectra of compound 3m 41
Figure S 62 ^{13}C NMR spectra of compound 3m 41
Figure S 63 MS spectra of compound 3m 42
Figure S 64 The purity of 3m from HPLC 43
Figure S 65 The purity of 3m from FTIR 43
Figure S 66 ^{1}H NMR spectra of compound 3n 44
Figure S 67 ^{13}C NMR spectra of compound 3n 44
Figure S 68 MS spectra of compound 3n 45
Figure S 69 The purity of 3n from HPLC 46
Figure S 70 The purity of 3n from FTIR 46

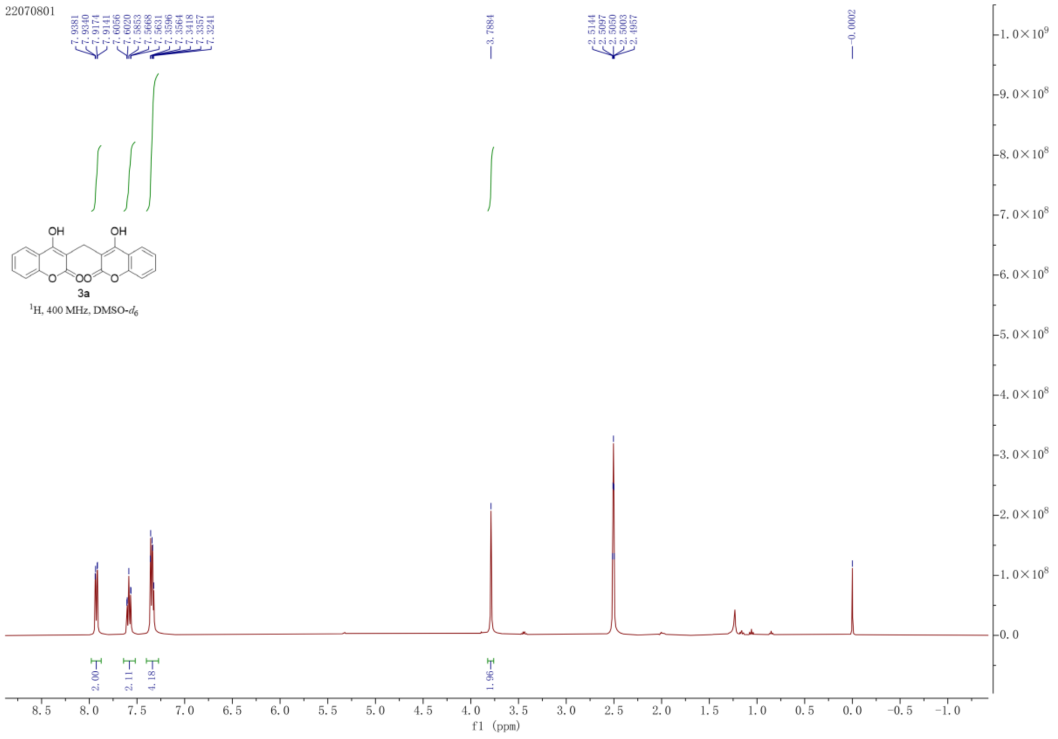

Figure S 1 ^{1}H NMR spectra of compound **3a**

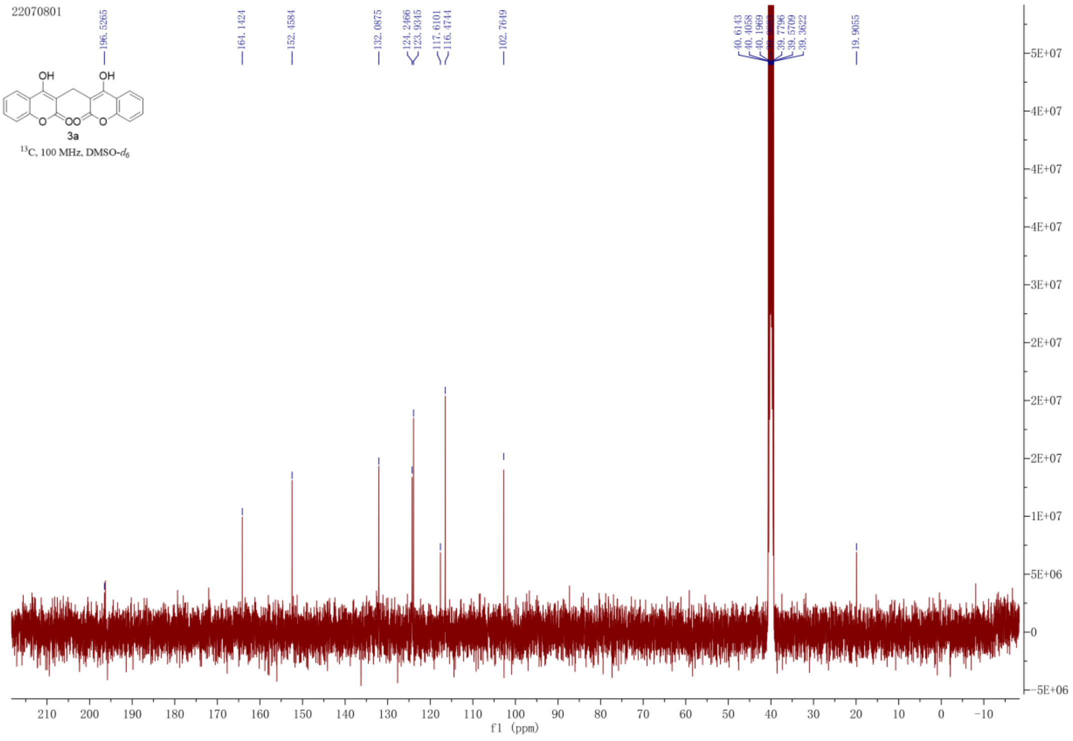

Figure S 2 ^{13}C NMR spectra of compound **3a**

Qualitative Analysis Report

**Data Filename**	12-Dicoumarolum-1.d	**Sample Name**	ZDJ
**Sample Type**	Sample	**Position**	P1-A1
**Instrument Name**	Instrument 1	**User Name**	
**Acq Method**	test.m	**Acquired Time**	11/6/2023 4:27:32 PM
**IRM Calibration Status**	Success	**DA Method**	Default.m
**Comment**			

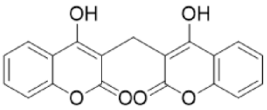

Chemical Formula: $C_{19}H_{12}O_6$
Exact Mass: 336.0634

Sample Group **Info.**

User Spectra

Fragmentor Voltage	Collision Energy	Ionization Mode
180	0	ESI

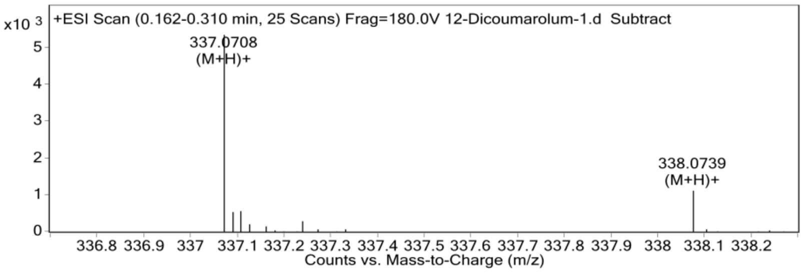

Formula Calculator Element Limits

Element	Min	Max
C	3	60
H	0	120
O	0	30
N	0	30

Formula Calculator Results

Formula	Best	Mass	Tgt Mass	Diff (ppm)	Ion Species	Score
C19 H12 O6	TRUE	336.0635	336.0634	-0.27	C19 H13 O6	99.81
C20 H8 N4 O2		336.0635	336.0647	3.7	C20 H9 N4 O2	94.02
C15 H8 N6 O4		336.0635	336.0607	-8.28	C15 H9 N6 O4	79.18

--- End Of Report ---

Figure S 3 MS spectra of compound **3a**

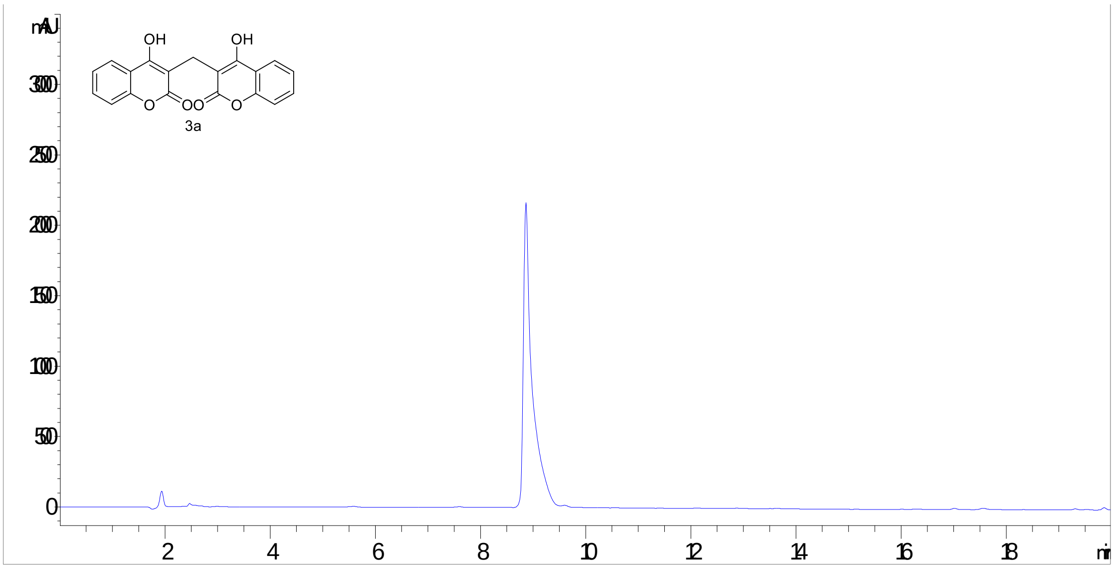

Figure S 4 The purity of **3a** from HPLC

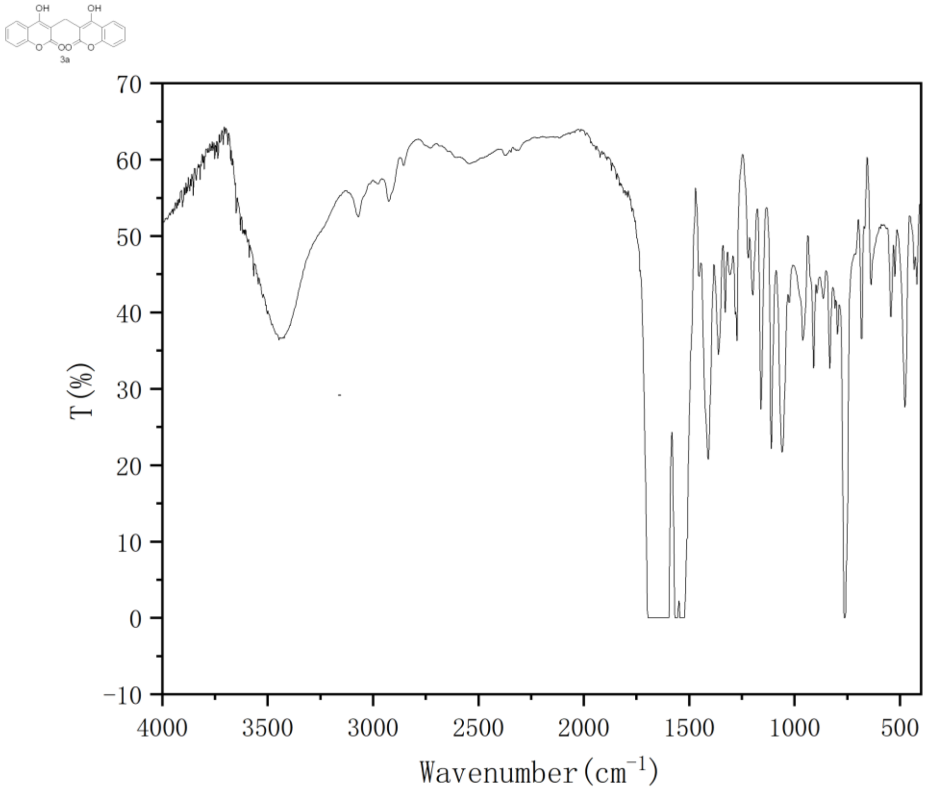

Figure S 5 The purity of **3a** from FTIR

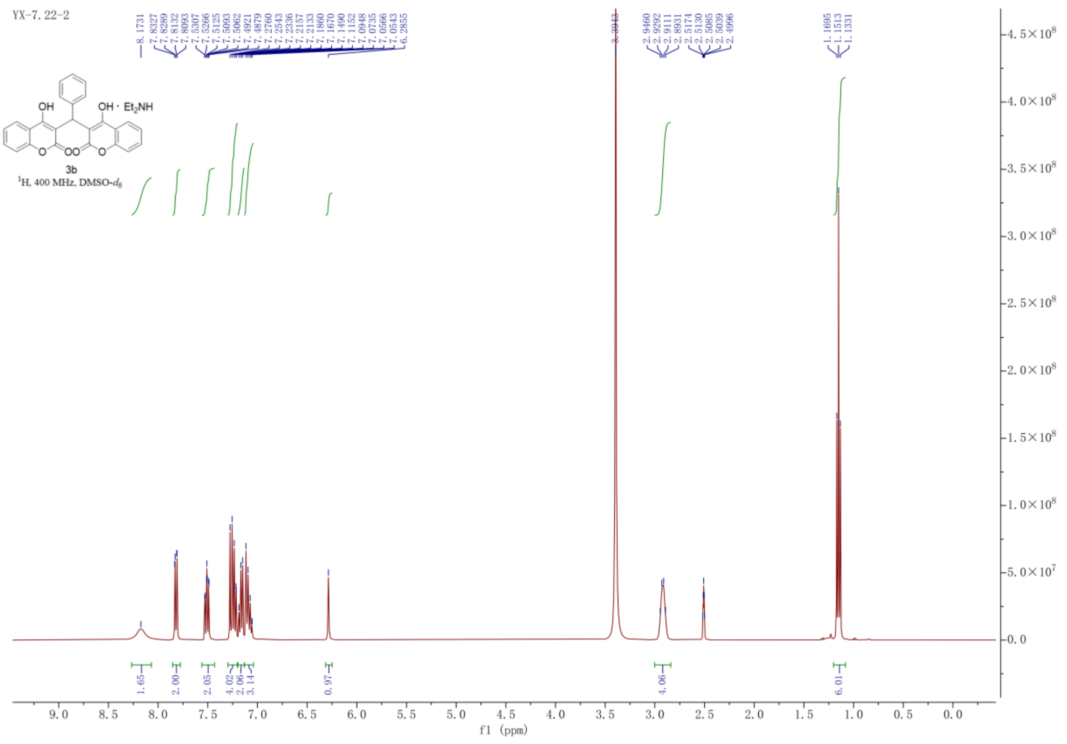

Figure S 6 ¹H NMR spectra of compound **3b**

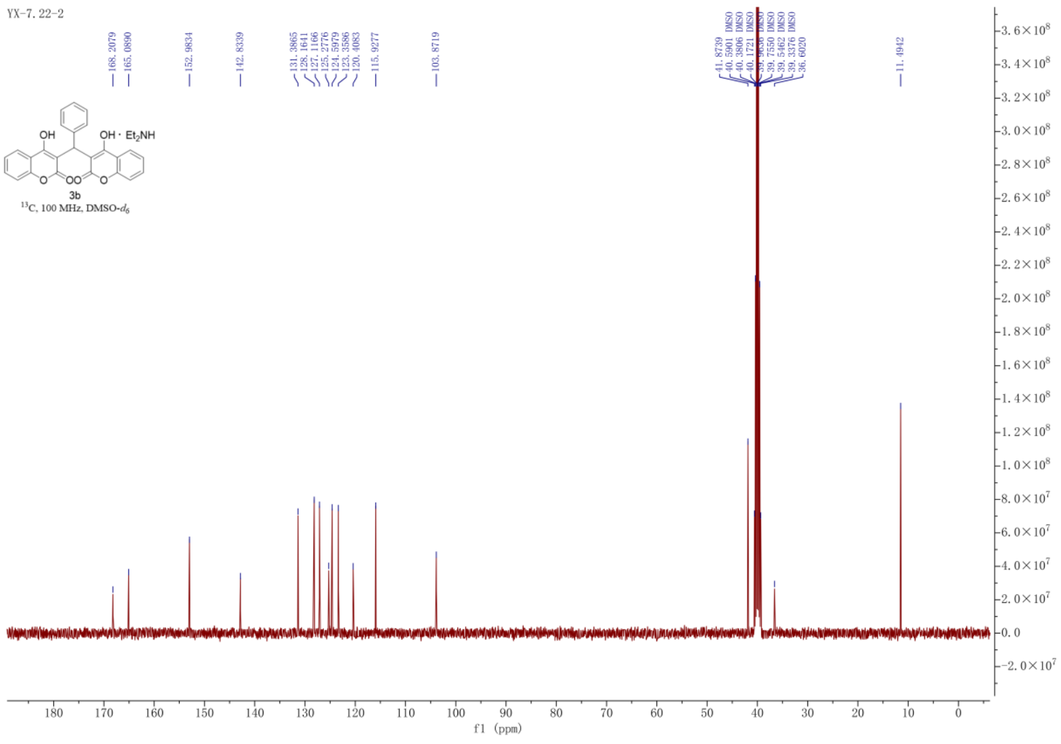

Figure S 7 ¹³C NMR spectra of compound **3b**

Qualitative Analysis Report

**Data Filename**	13-Dicoumarolum-2.d	**Sample Name**	ZDJ
**Sample Type**	Sample	**Position**	P1-A2
**Instrument Name**	Instrument 1	**User Name**	
**Acq Method**	test.m	**Acquired Time**	11/6/2023 4:28:44 PM
**IRM Calibration Status**	Success	**DA Method**	Default.m
**Comment**			

Sample Group **Info.**

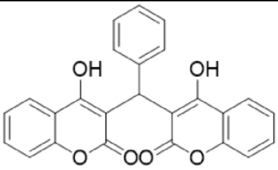

Chemical Formula: $C_{25}H_{16}O_6$
Exact Mass: 412.0947

User Spectra

Fragmentor Voltage	Collision Energy	Ionization Mode
180	0	ESI

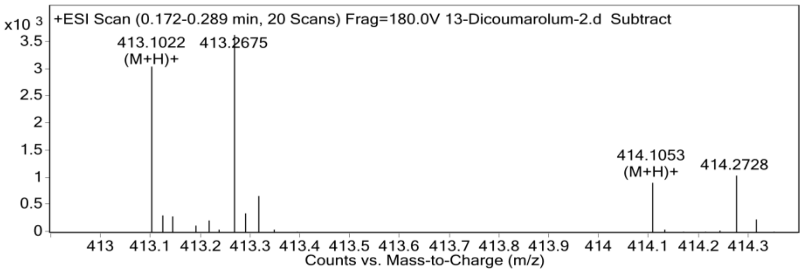

Formula Calculator Element Limits

Element	Min	Max
C	3	60
H	0	120
O	0	30
N	0	30

Formula Calculator Results

Formula	Best	Mass	Tgt Mass	Diff (ppm)	Ion Species	Score
C25 H16 O6	TRUE	412.0949	412.0947	-0.46	C25 H17 O6	98.41
C26 H12 N4 O2		412.0949	412.096	2.76	C26 H13 N4 O2	95.67
C21 H12 N6 O4		412.0949	412.092	-7.01	C21 H13 N6 O4	78.34
C13 H20 N2 O13		412.0949	412.0965	4.01	C13 H21 N2 O13	71.49
C14 H16 N6 O9		412.0949	412.0979	7.22	C14 H17 N6 O9	63.59

--- End Of Report ---

Agilent Technologies Page 1 of 1 Printed at: 4:44 PM on: 11/6/2023

Figure S 8 MS spectra of compound **3b**

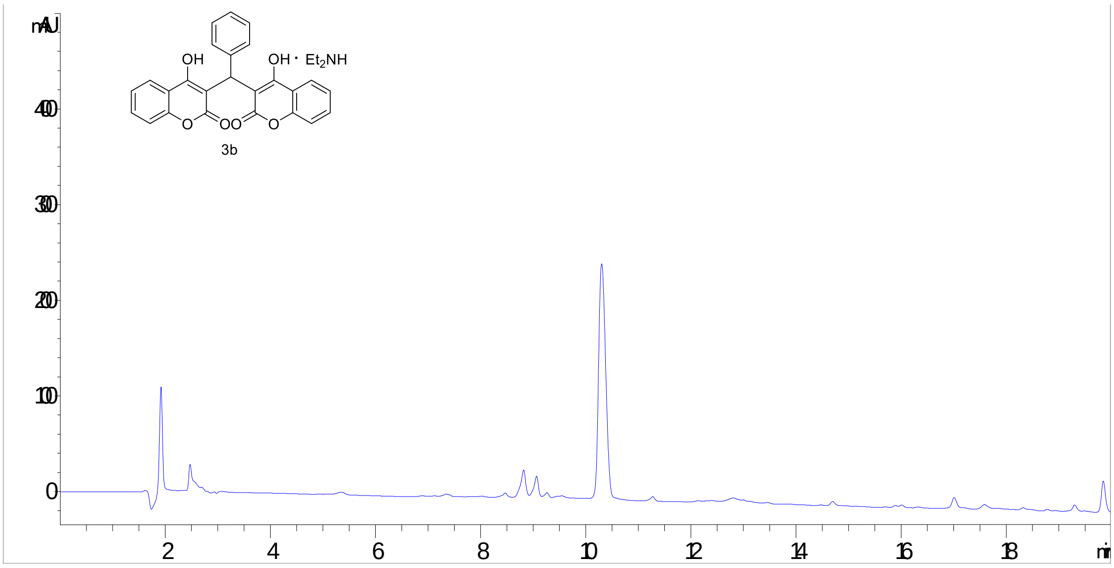

Figure S 9 The purity of **3b** from HPLC

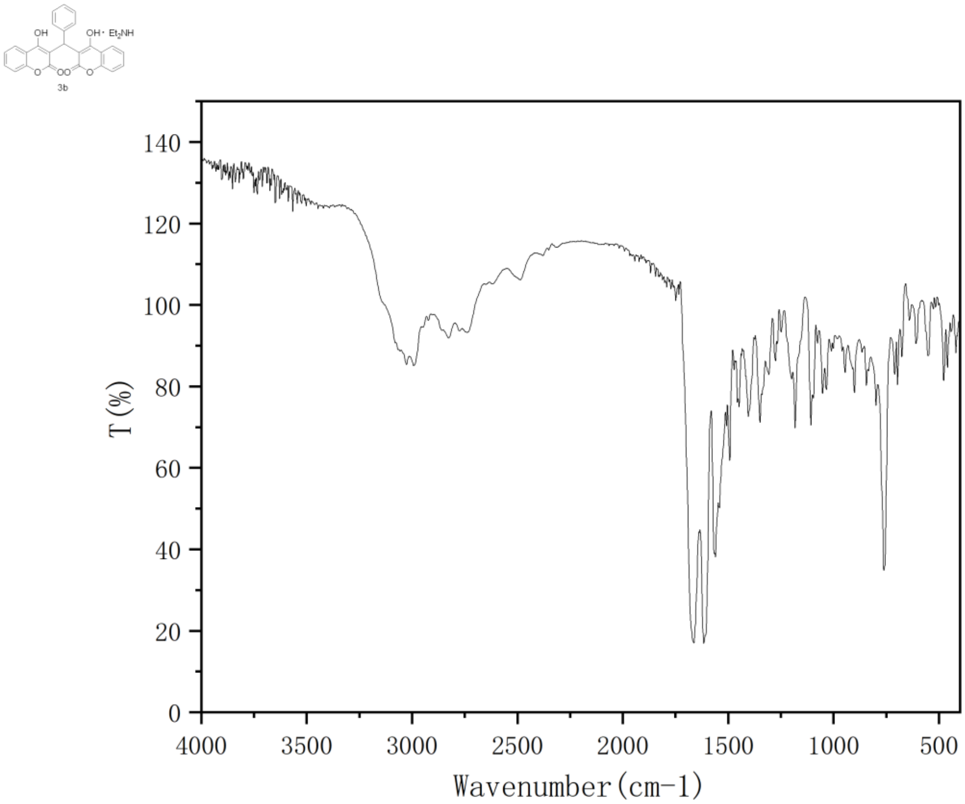

Figure S 10 The purity of **3b** from FTIR

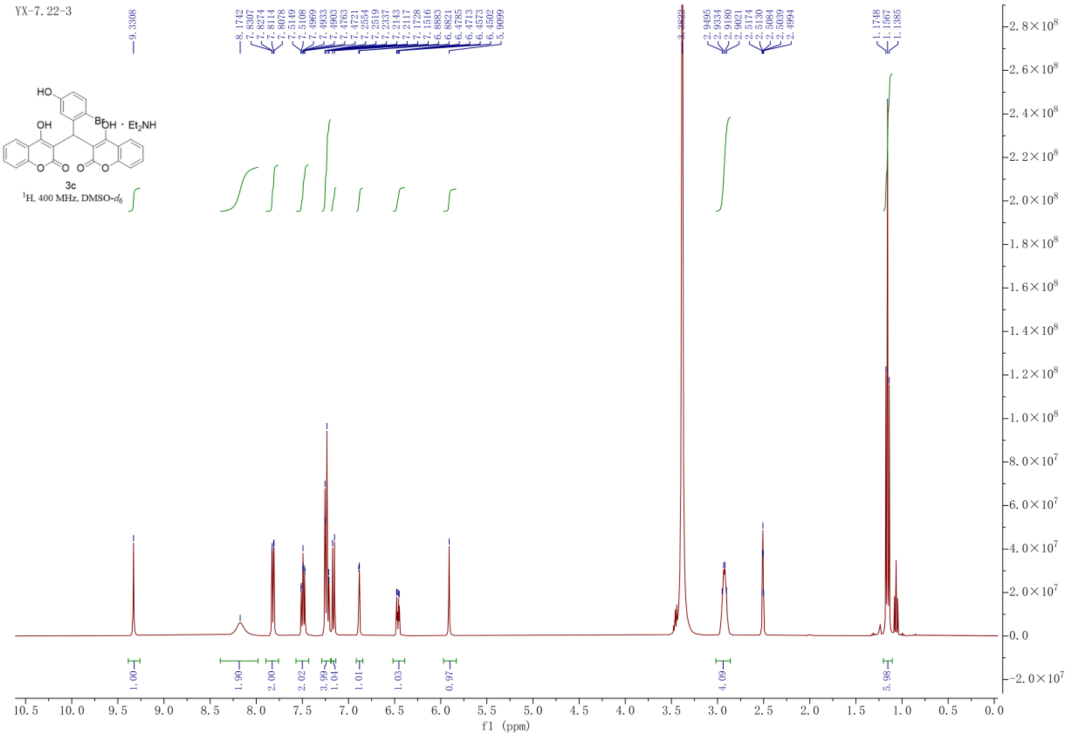

Figure S 11 ^{1}H NMR spectra of compound **3c**

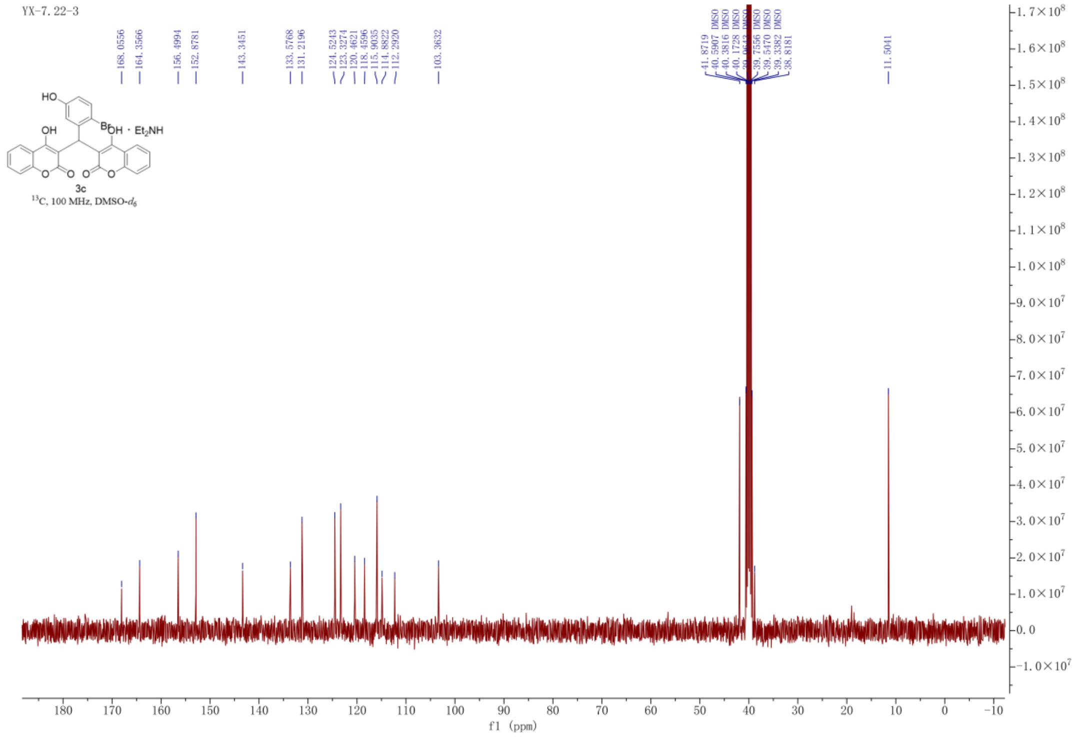

Figure S 12 ^{13}C NMR spectra of compound **3c**

Qualitative Analysis Report

**Data Filename**	14-Dicoumarolum-3.d	**Sample Name**	ZDJ
**Sample Type**	Sample	**Position**	P1-A3
**Instrument Name**	Instrument 1	**User Name**	
**Acq Method**	test.m	**Acquired Time**	11/6/2023 4:29:57 PM
**IRM Calibration Status**	Success	**DA Method**	Default.m
**Comment**			

Sample Group **Info.**

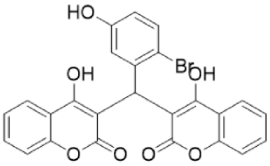

Chemical Formula: $C_{25}H_{15}BrO_7$
Exact Mass: 506.0001

User Spectra

Fragmentor Voltage	Collision Energy	Ionization Mode
180	0	ESI

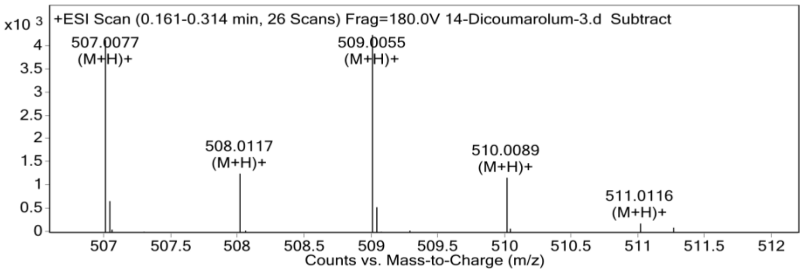

Formula Calculator Element Limits

Element	Min	Max
C	3	60
H	0	120
O	0	30
N	0	30
Br	0	5

Formula Calculator Results

Formula	Best	Mass	Tgt Mass	Diff (ppm)	Ion Species	Score
C25 H15 Br O7	TRUE	506.0001	506.0001	-0.06	C25 H16 Br O7	98.75
C26 H11 Br N4 O3		506.0002	506.0015	2.54	C26 H12 Br N4 O3	95.71
C21 H11 Br N6 O5		506.0002	505.9974	-5.5	C21 H12 Br N6 O5	82.41
C13 H19 Br N2 O14		506.0002	506.002	3.45	C13 H20 Br N2 O14	74.11
C20 H15 Br N2 O9		506.0002	505.9961	-8.1	C20 H16 Br N2 O9	68.93

--- End Of Report ---

Agilent Technologies Page 1 of 1 Printed at: 4:47 PM on: 11/6/2023

Figure S 13 MS spectra of compound **3c**

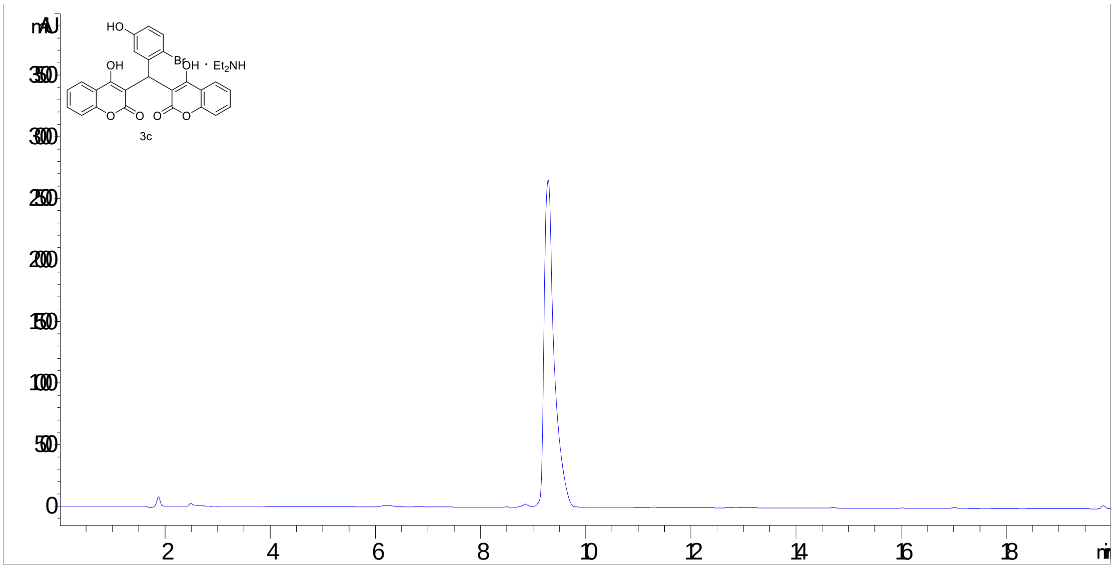

Figure S 14 The purity of **3c** from HPLC

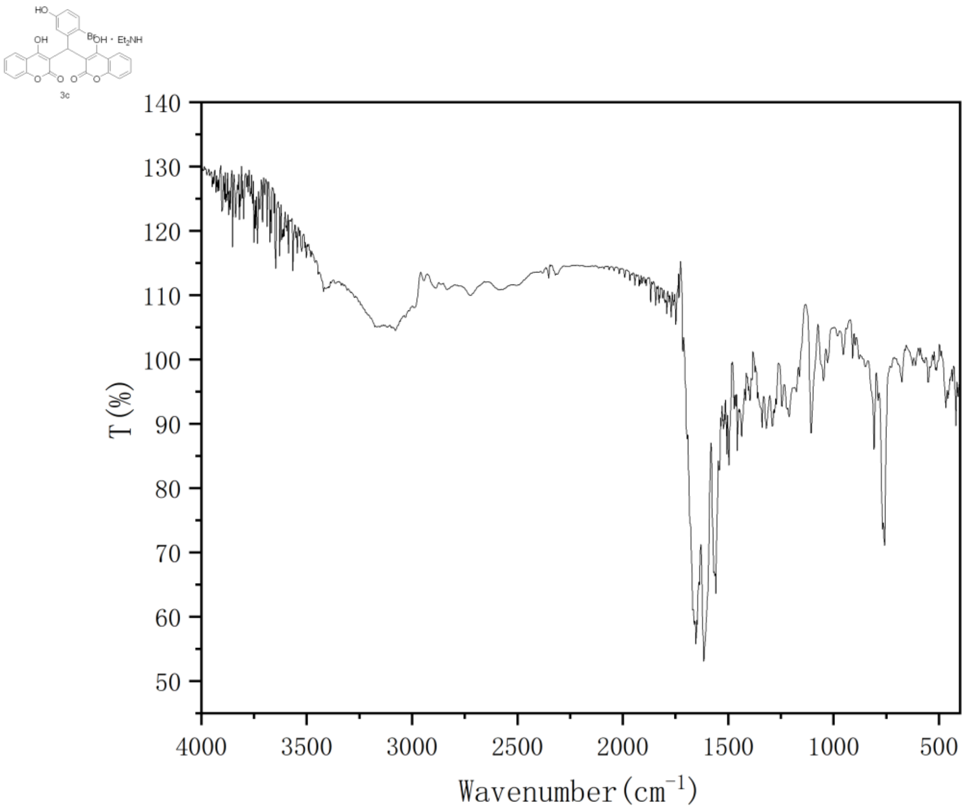

Figure S 15 The purity of **3c** from FTIR

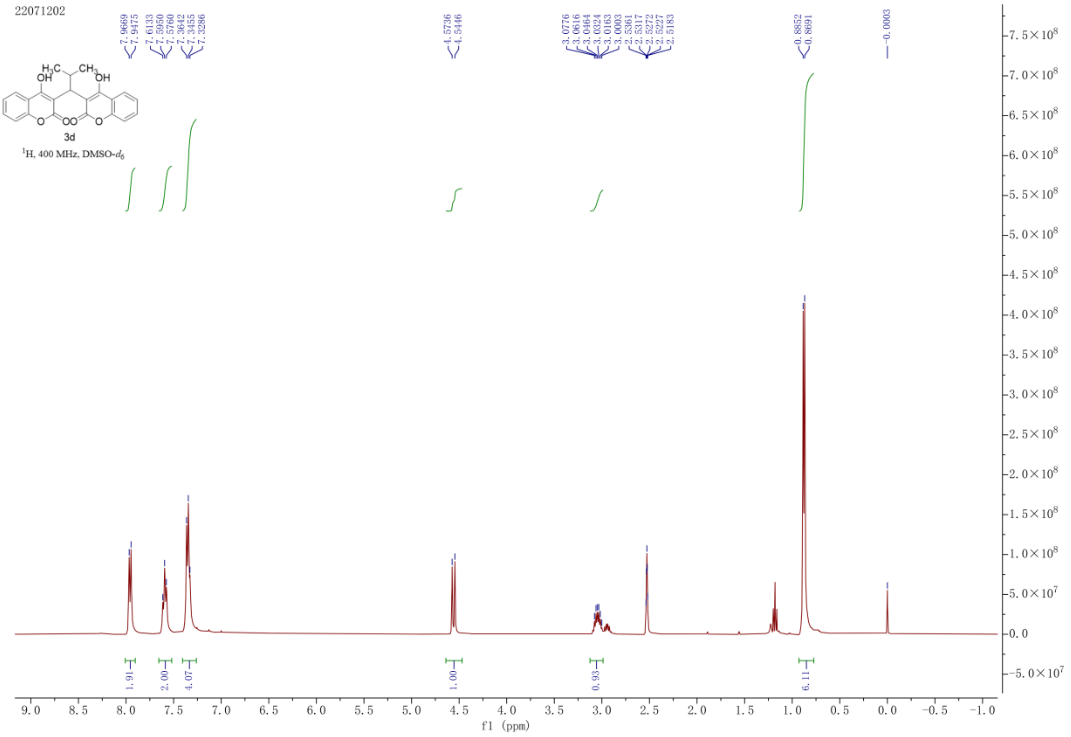

Figure S 16 ^{1}H NMR spectra of compound **3d**

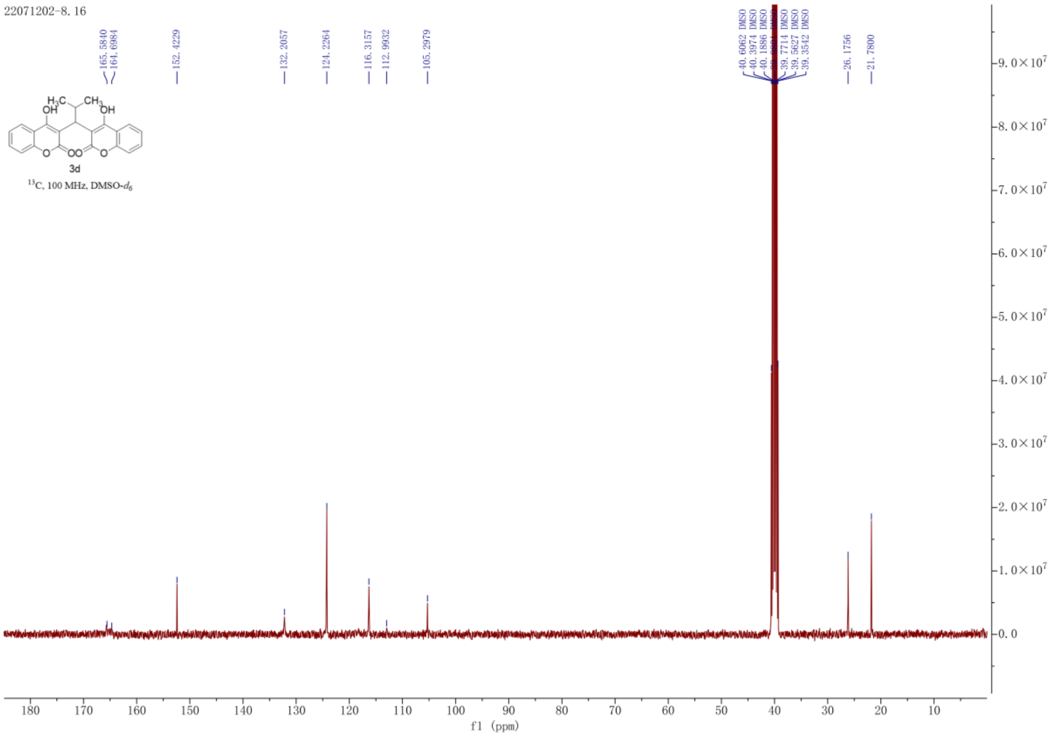

Figure S 17 ^{13}C NMR spectra of compound **3d**

Qualitative Analysis Report

**Data Filename**	15-Dicoumarolum-4.d	**Sample Name**	ZDJ
**Sample Type**	Sample	**Position**	P1-A4
**Instrument Name**	Instrument 1	**User Name**	
**Acq Method**	test.m	**Acquired Time**	11/6/2023 4:31:09 PM
**IRM Calibration Status**	Success	**DA Method**	Default.m
**Comment**			

Sample Group **Info.**

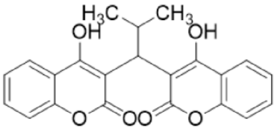

Chemical Formula: $C_{22}H_{18}O_6$
Exact Mass: 378.1103

User Spectra

Fragmentor Voltage	Collision Energy	Ionization Mode
180	0	ESI

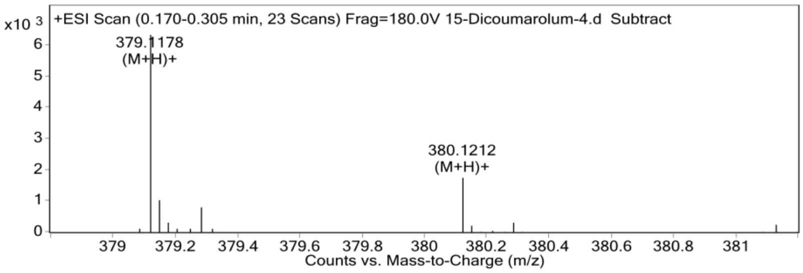

Formula Calculator Element Limits

Element	Min	Max
C	3	60
H	0	120
O	0	30
N	0	30

Formula Calculator Results

Formula	Best	Mass	Tgt Mass	Diff (ppm)	Ion Species	Score
C22 H18 O6	TRUE	378.1105	378.1103	-0.46	C22 H19 O6	97.36
C23 H14 N4 O2		378.1105	378.1117	3.06	C23 H15 N4 O2	95.9
C19 H10 N10		378.1105	378.109	-4.08	C19 H11 N10	90.34
C18 H14 N6 O4		378.1105	378.1077	-7.6	C18 H15 N6 O4	75.23

--- End Of Report ---

Figure S 18 MS spectra of compound **3d**

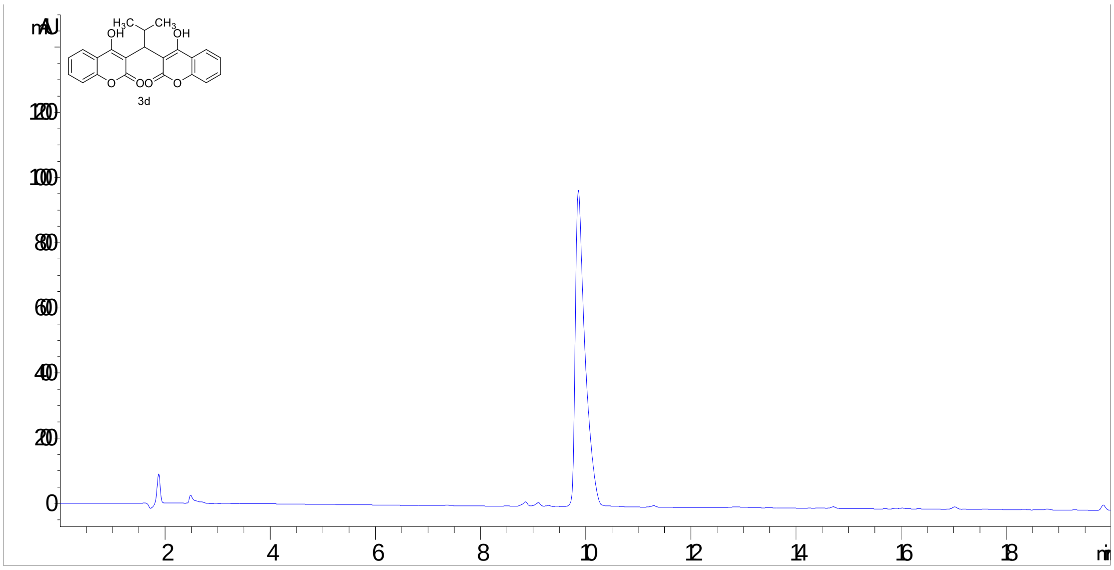

Figure S 19 The purity of **3d** from HPLC

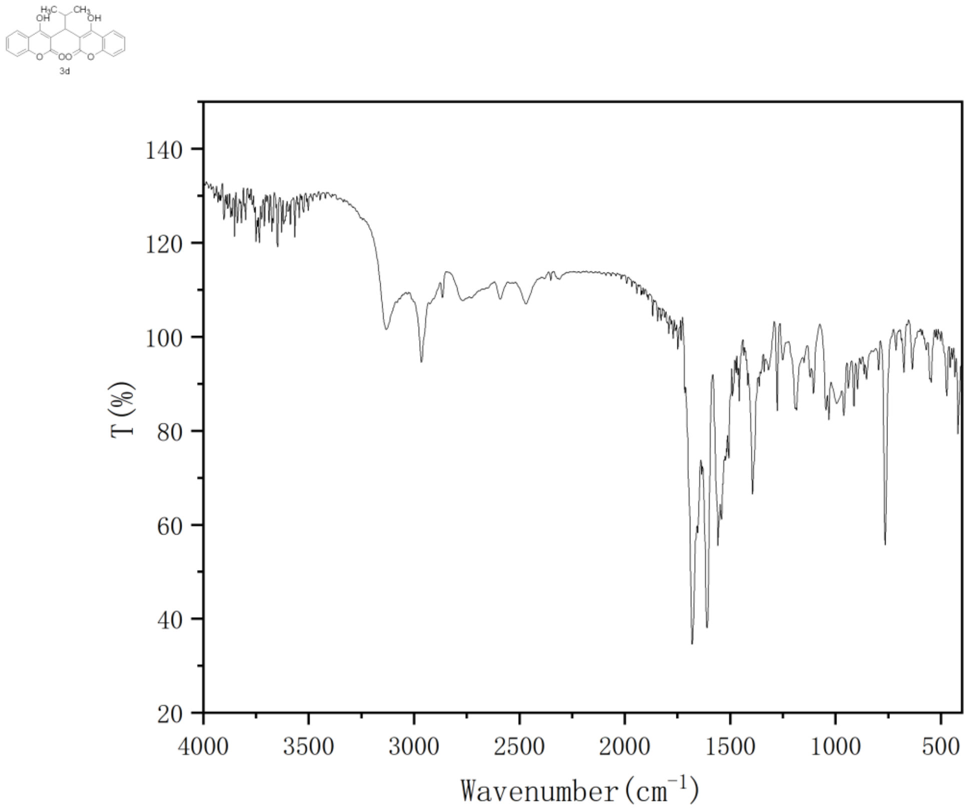

Figure S 20 The purity of **3d** from FTIR

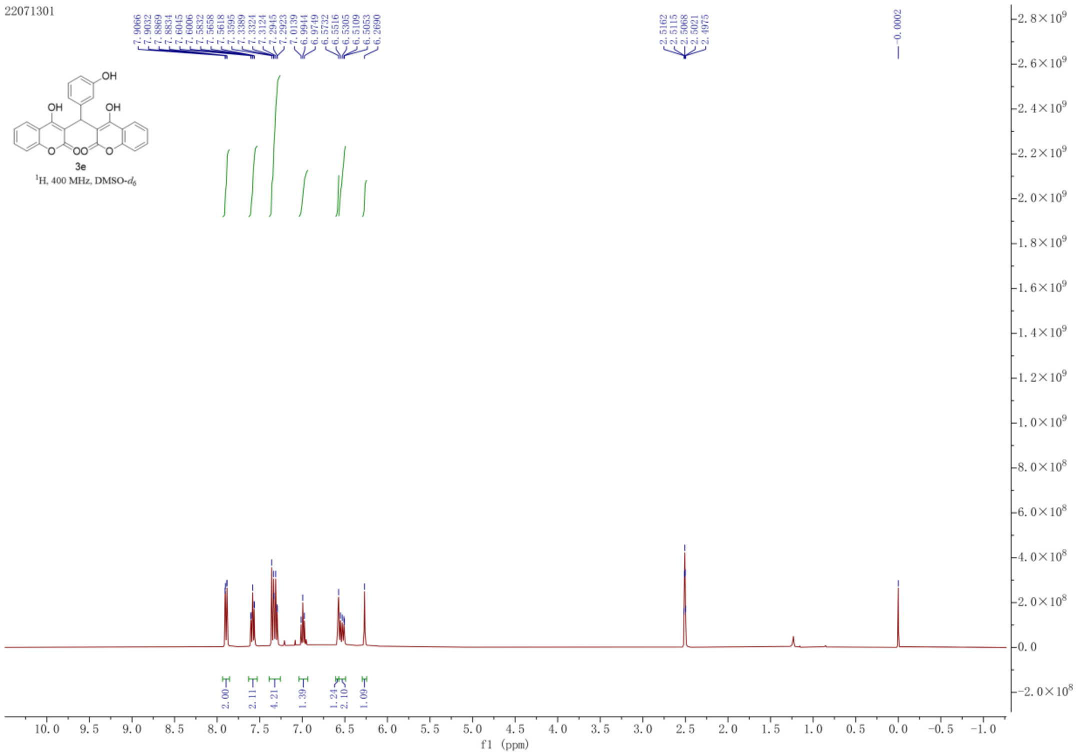

Figure S 21 ^{1}H NMR spectra of compound **3e**

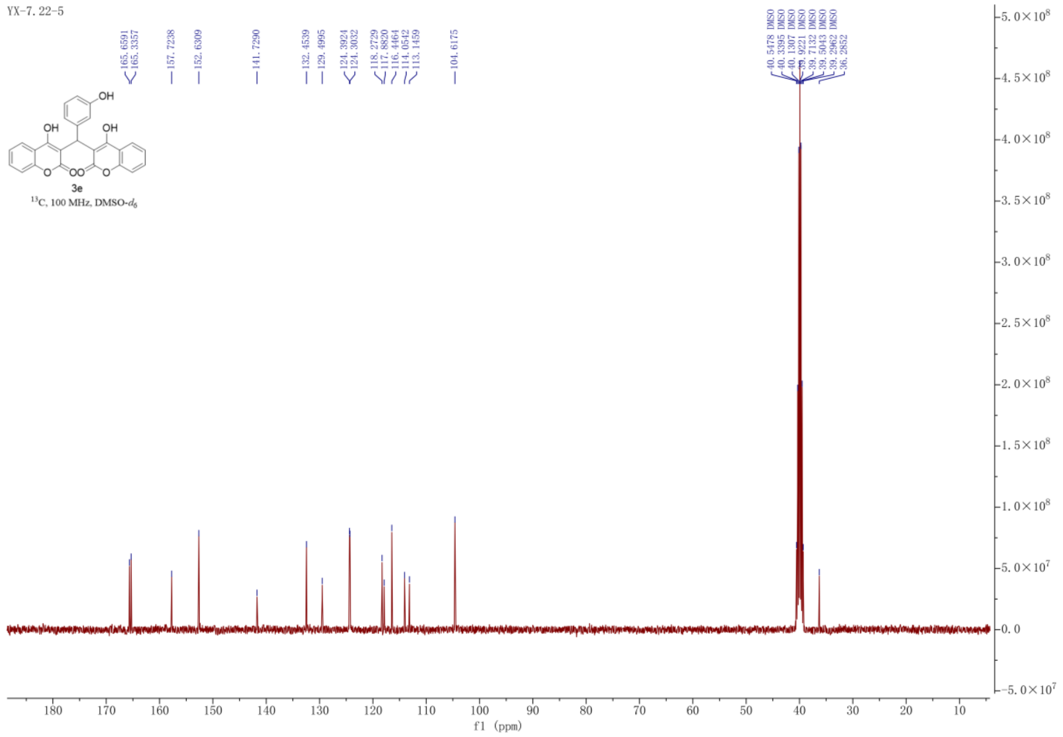

Figure S 22 ^{13}C NMR spectra of compound **3e**

Qualitative Analysis Report

**Data Filename**	16-Dicoumarolum-5.d	**Sample Name**	ZDJ
**Sample Type**	Sample	**Position**	P1-A5
**Instrument Name**	Instrument 1	**User Name**	
**Acq Method**	test.m	**Acquired Time**	11/6/2023 4:32:22 PM
**IRM Calibration Status**	Success	**DA Method**	Default.m
**Comment**			

Sample Group **Info.**

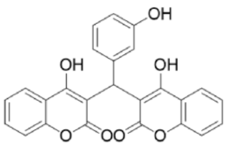

Chemical Formula: $C_{25}H_{16}O_7$
Exact Mass: 428.0896

User Spectra

Fragmentor Voltage	Collision Energy	Ionization Mode
180	0	ESI

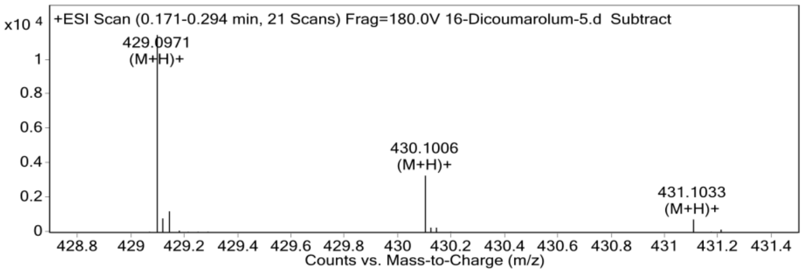

Formula Calculator Element Limits

Element	Min	Max
C	3	60
H	0	120
O	0	30
N	0	30

Formula Calculator Results

Formula	Best	Mass	Tgt Mass	Diff (ppm)	Ion Species	Score
C25 H16 O7	TRUE	428.0898	428.0896	-0.46	C25 H17 O7	99.04
C26 H12 N4 O3		428.0898	428.0909	2.65	C26 H13 N4 O3	95.67
C21 H12 N6 O5		428.0898	428.0869	-6.76	C21 H13 N6 O5	79.61
C13 H20 N2 O14		428.0898	428.0915	3.85	C13 H21 N2 O14	72.61

--- End Of Report ---

Figure S 23 MS spectra of compound **3e**

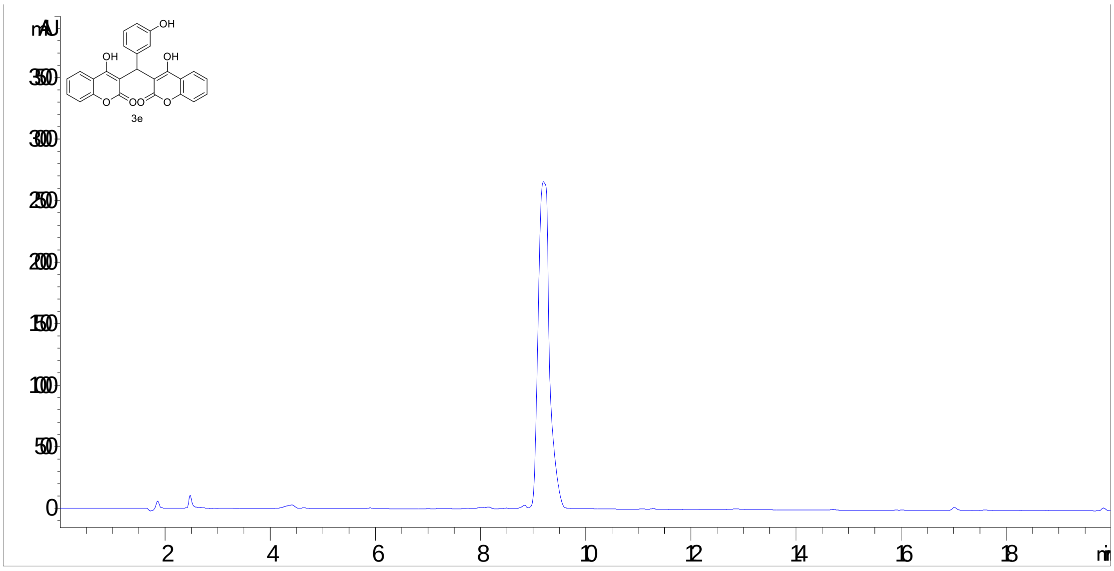

Figure S 24 The purity of **3e** from HPLC

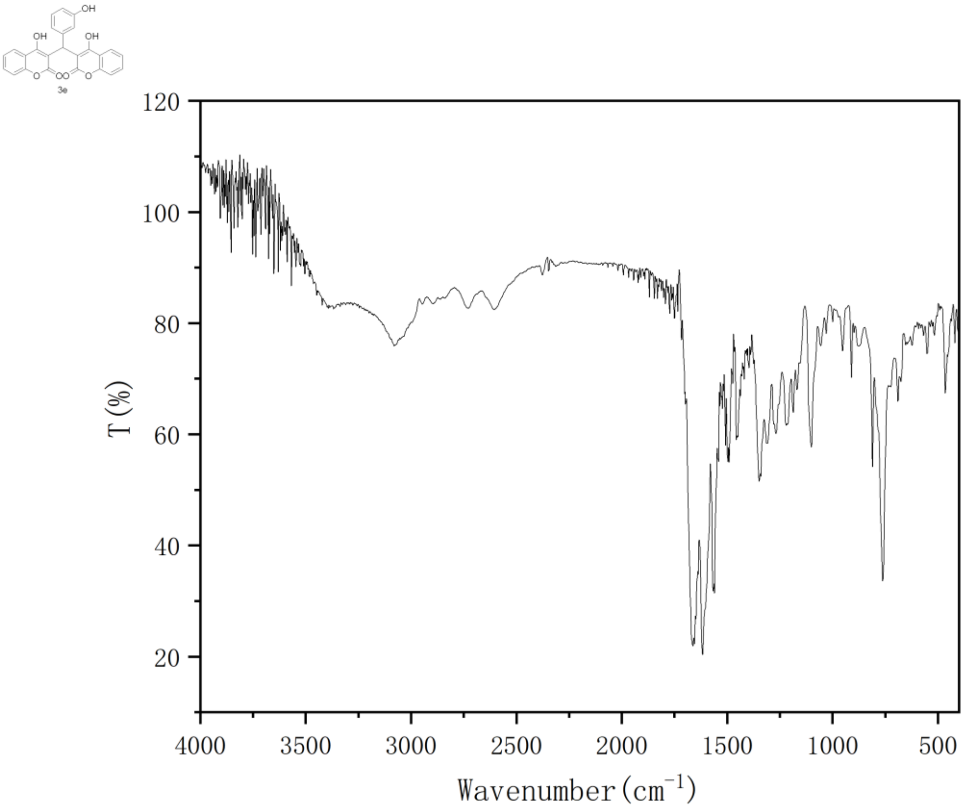

Figure S 25 The purity of **3e** from FTIR

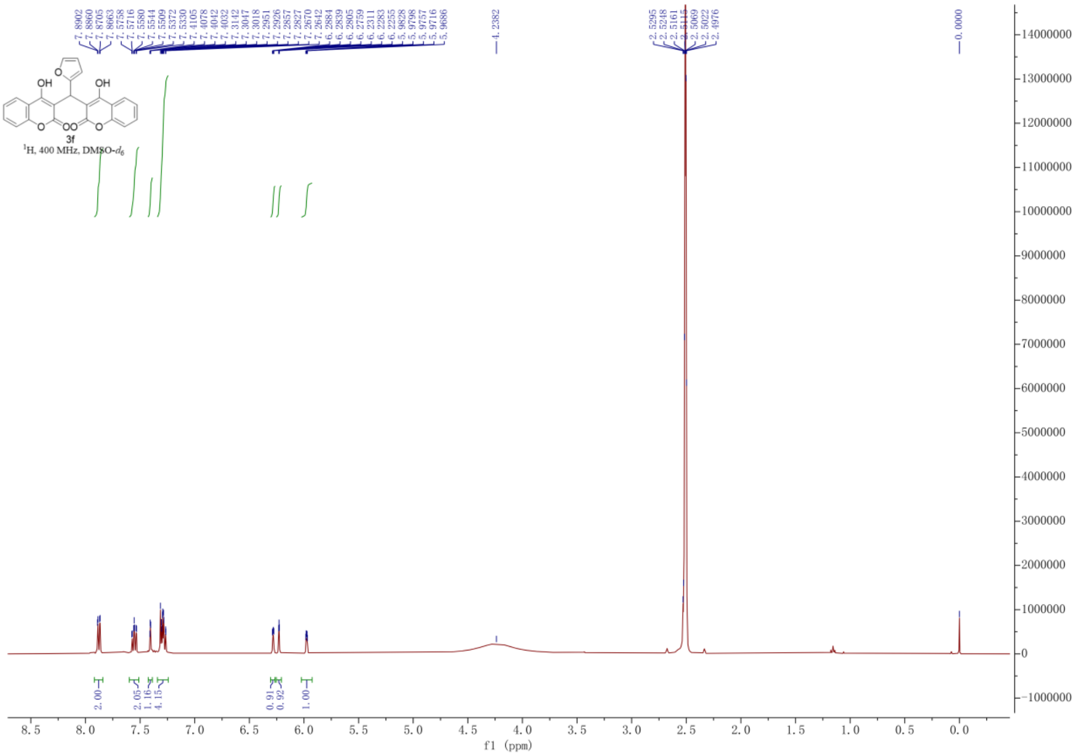

Figure S 26 ^{1}H NMR spectra of compound **3f**

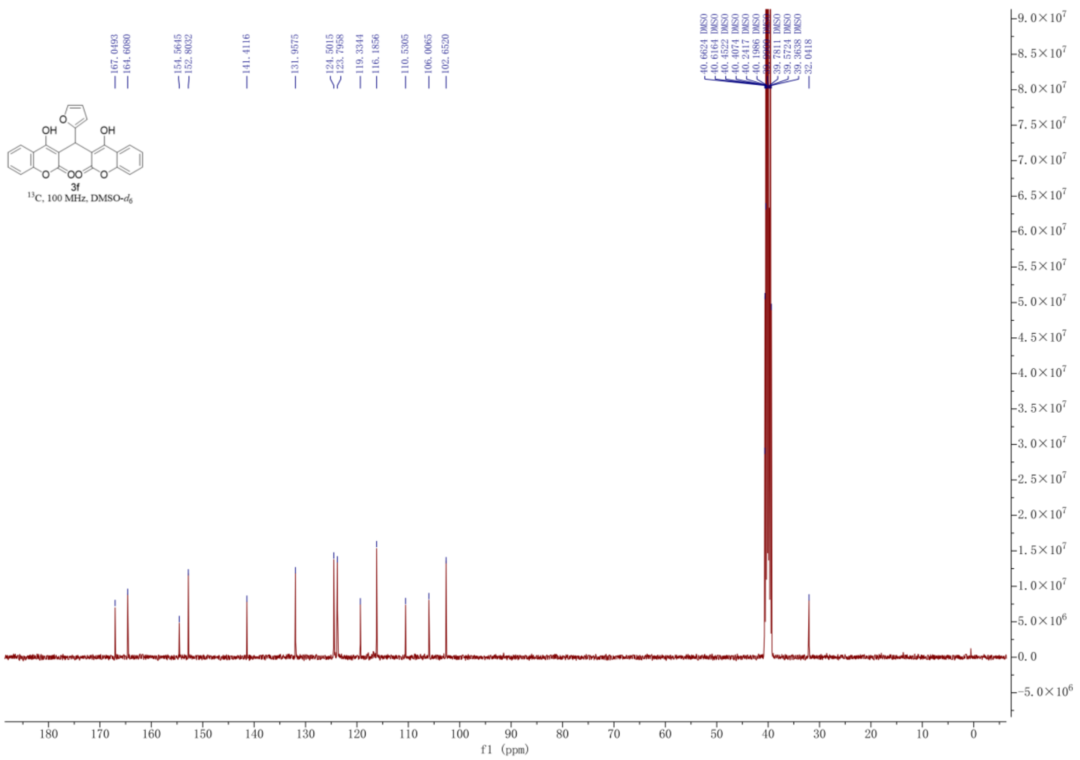

Figure S 27 ^{13}C NMR spectra of compound **3f**

Qualitative Analysis Report

**Data Filename**	18-Dicoumarolum-7.d	**Sample Name**	ZDJ
**Sample Type**	Sample	**Position**	P1-A7
**Instrument Name**	Instrument 1	**User Name**	
**Acq Method**	test.m	**Acquired Time**	11/6/2023 4:34:46 PM
**IRM Calibration Status**	Success	**DA Method**	Default.m
**Comment**			
**Sample Group**	**Info.**		

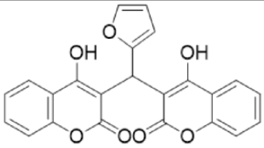

Chemical Formula: $C_{23}H_{14}O_7$
Exact Mass: 402.0740

User Spectra

Fragmentor Voltage	Collision Energy	Ionization Mode
180	0	ESI

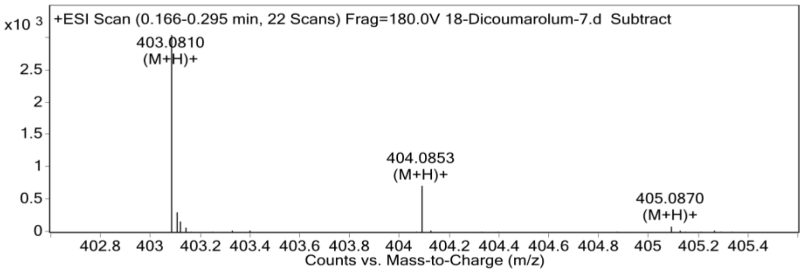

Formula Calculator Element Limits

Element	Min	Max
C	3	60
H	0	120
O	0	30

Formula Calculator Results

Formula	Best	Mass	Tgt Mass	Diff (ppm)	Ion Species	Score
C23 H14 O7	TRUE	402.0737	402.074	0.55	C23 H15 O7	98.25

--- End Of Report ---

Figure S 28 MS spectra of compound **3f**

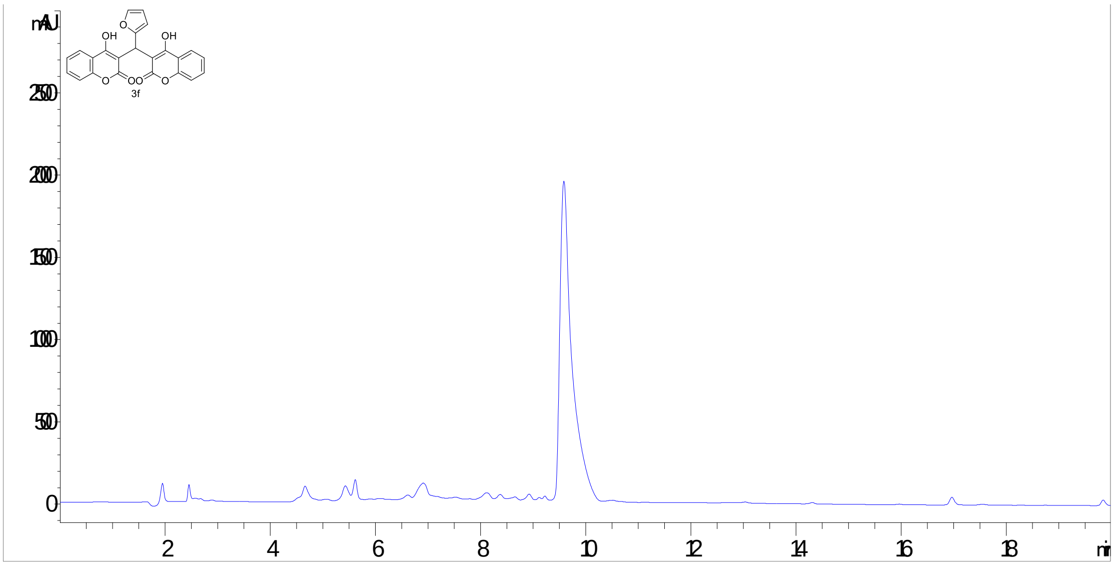

Figure S 29 The purity of **3f** from HPLC

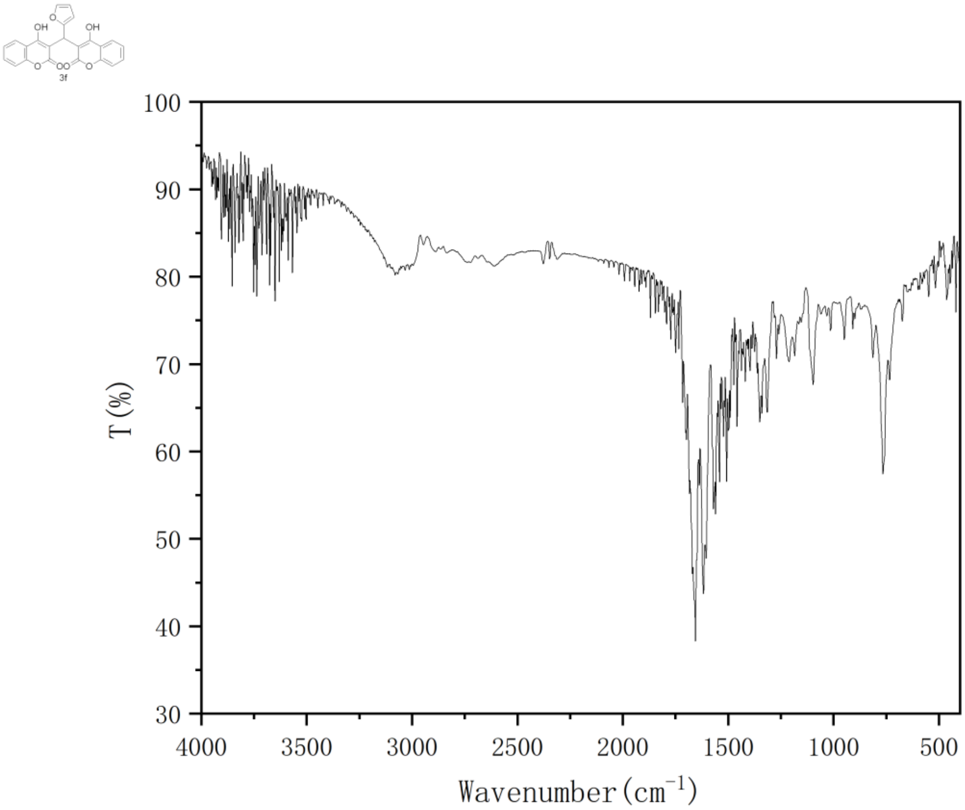

Figure S 30 The purity of **3f** from FTIR

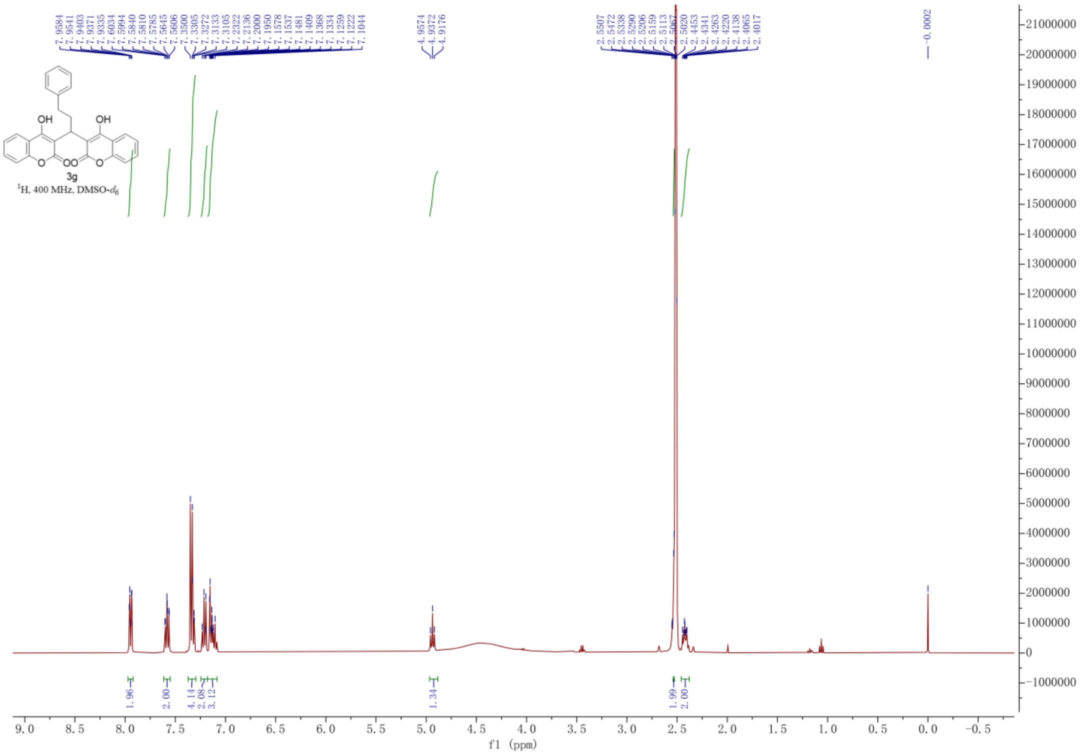

Figure S 31 ^{1}H NMR spectra of compound **3g**

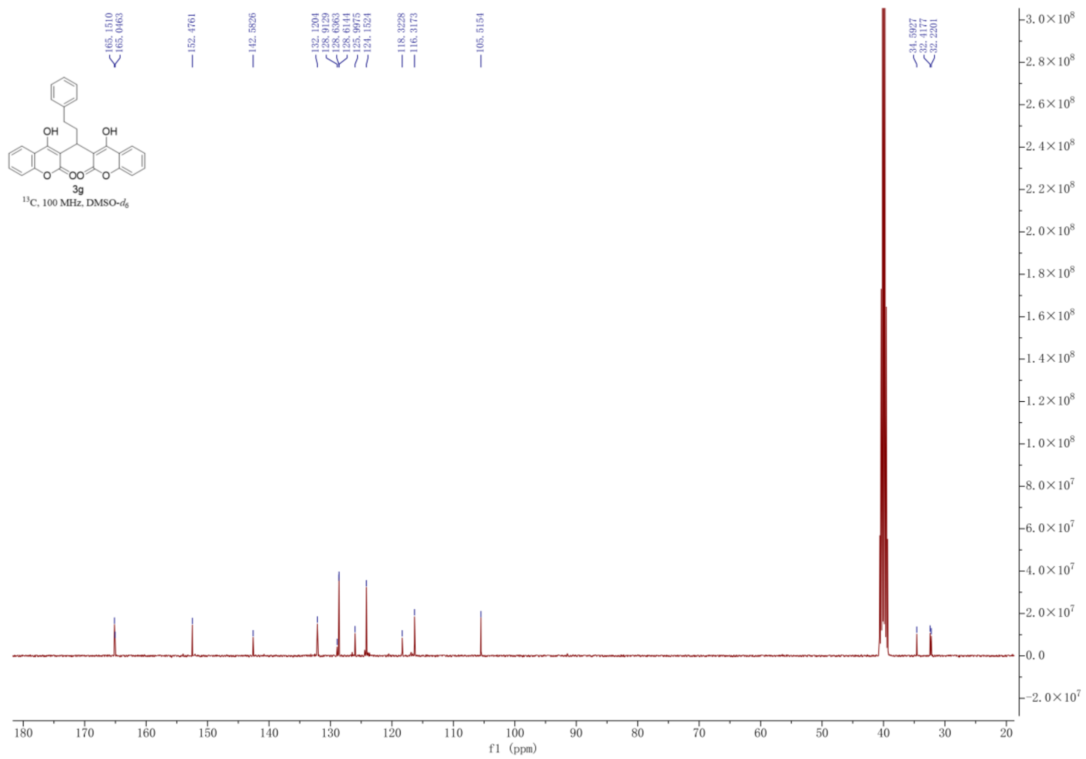

Figure S 32 ^{13}C NMR spectra of compound **3g**

Qualitative Analysis Report

**Data Filename**	19-Dicoumarolum-8.d	**Sample Name**	ZDJ
**Sample Type**	Sample	**Position**	P1-A8
**Instrument Name**	Instrument 1	**User Name**	
**Acq Method**	test.m	**Acquired Time**	11/6/2023 4:35:58 PM
**IRM Calibration Status**	Success	**DA Method**	Default.m
**Comment**			

Sample Group **Info.**

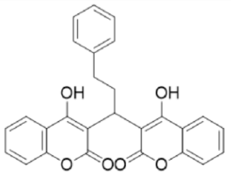

Chemical Formula: $C_{27}H_{20}O_6$
Exact Mass: 440.1260

User Spectra

Fragmentor Voltage	Collision Energy	Ionization Mode
180	0	ESI

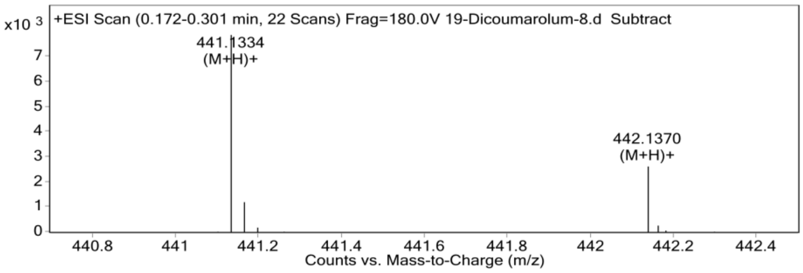

Formula Calculator Element Limits

Element	Min	Max
C	3	60
H	0	120
O	0	30

Formula Calculator Results

Formula	Best	Mass	Tgt Mass	Diff (ppm)	Ion Species	Score
C27 H20 O6	TRUE	440.1262	440.126	-0.38	C27 H21 O6	97.06

--- End Of Report ---

Figure S 33 MS spectra of compound **3g**

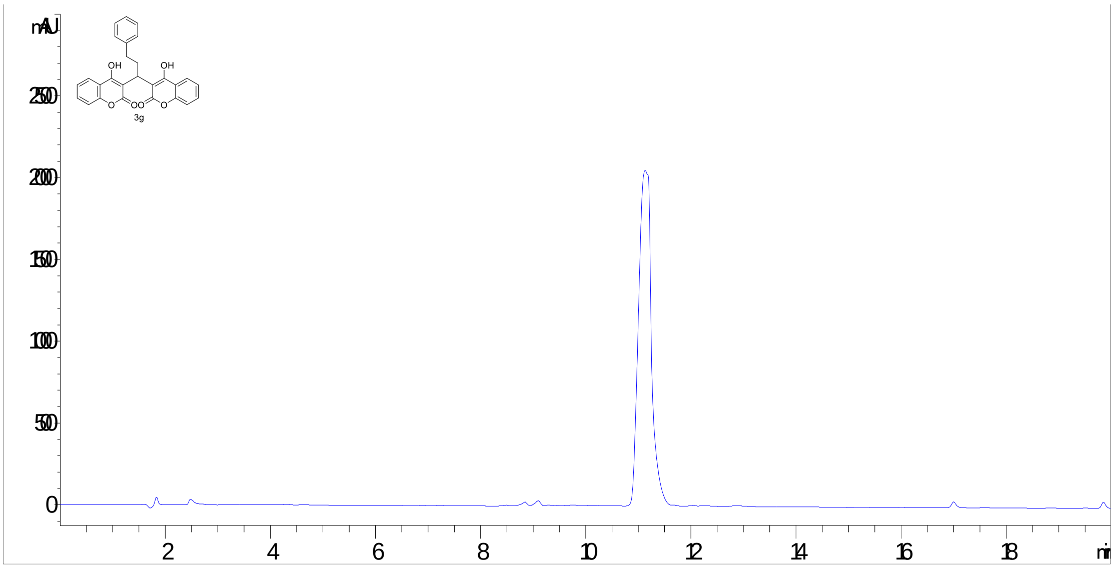

Figure S 34 The purity of **3g** from HPLC

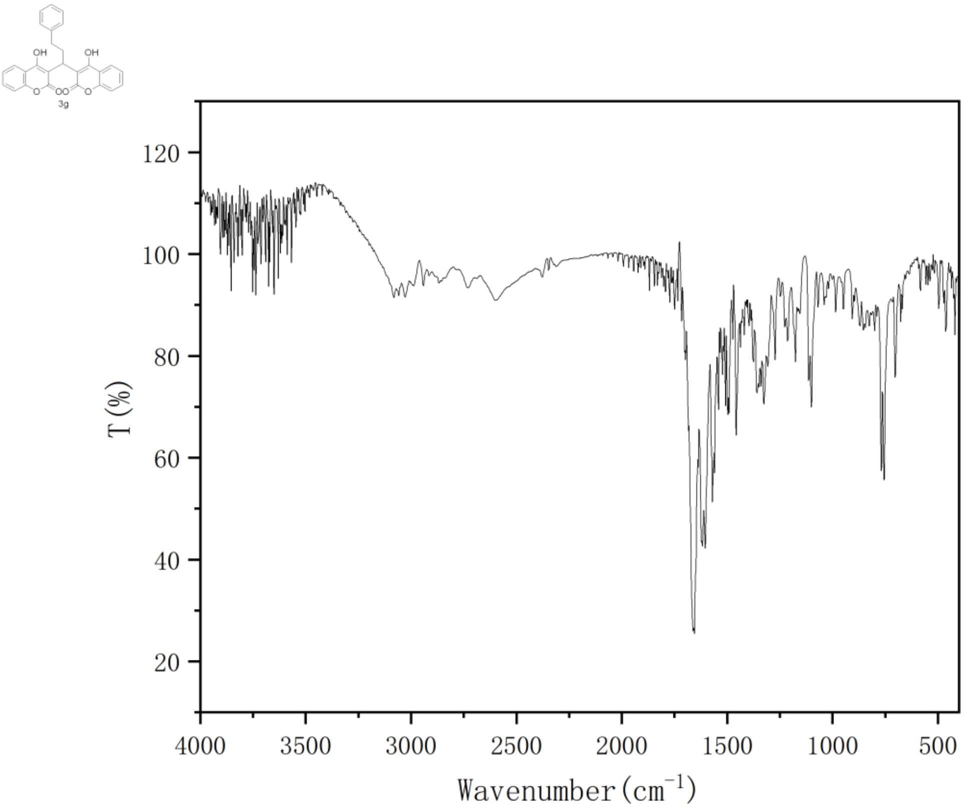

Figure S 35 The purity of **3g** from FTIR

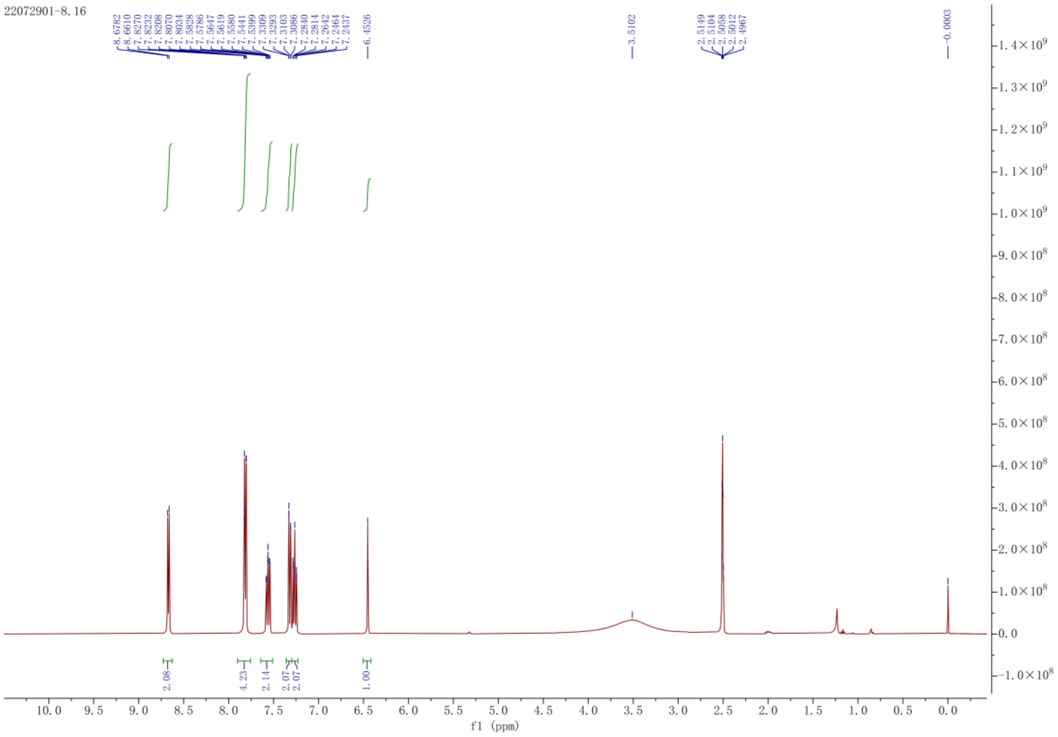

Figure S 36 ^{1}H NMR spectra of compound **3h**

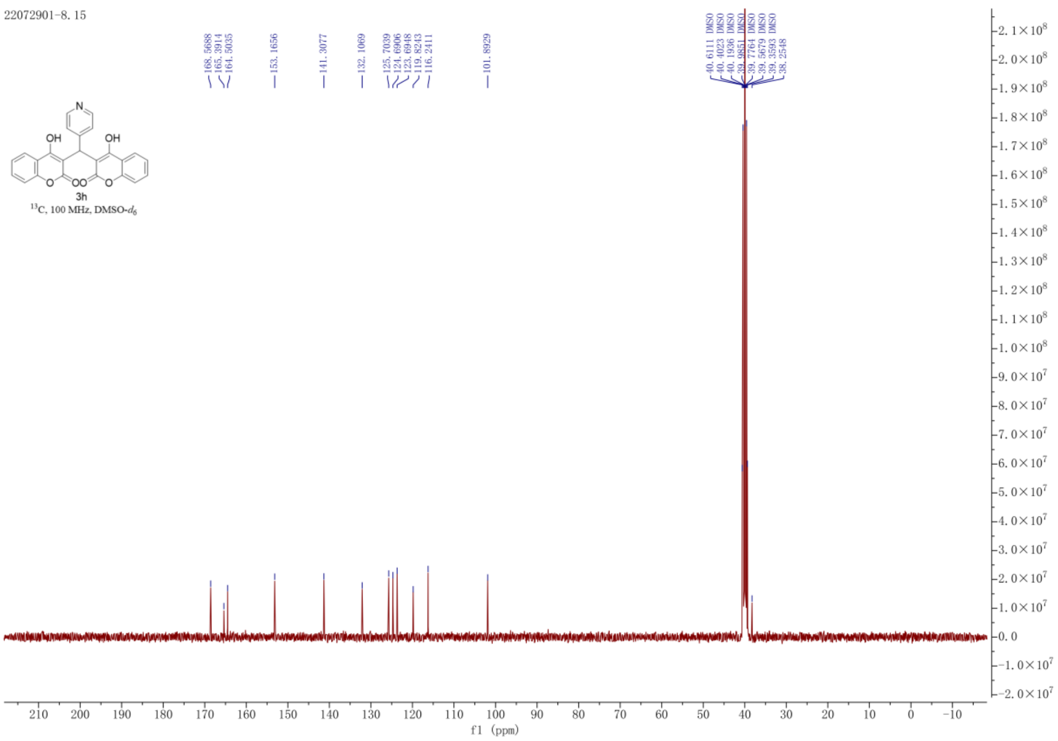

Figure S 37 ^{13}C NMR spectra of compound **3h**

Qualitative Analysis Report

**Data Filename**	20-Dicoumarolum-9.d	**Sample Name**	ZDJ
**Sample Type**	Sample	**Position**	P1-A9
**Instrument Name**	Instrument 1	**User Name**	
**Acq Method**	test.m	**Acquired Time**	11/6/2023 4:37:09 PM
**IRM Calibration Status**	Success	**DA Method**	Default.m
**Comment**			

Sample Group **Info.**

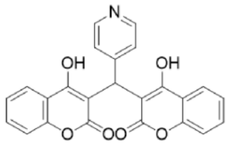

Chemical Formula: $C_{24}H_{15}NO_6$
Exact Mass: 413.0899

User Spectra

Fragmentor Voltage	Collision Energy	Ionization Mode
180	0	ESI

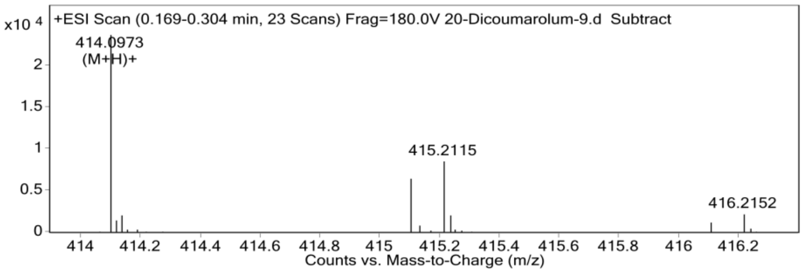

Formula Calculator Element Limits

Element	Min	Max
C	3	60
H	0	120
O	0	30
N	0	10

Formula Calculator Results

Formula	Best	Mass	Tgt Mass	Diff (ppm)	Ion Species	Score
C24 H15 N O6	TRUE	413.09	413.0899	-0.24	C24 H16 N O6	99.58
C25 H11 N5 O2		413.09	413.0913	2.98	C25 H12 N5 O2	95.04
C20 H11 N7 O4		413.09	413.0873	-6.77	C20 H12 N7 O4	81.22
C12 H19 N3 O13		413.09	413.0918	4.22	C12 H20 N3 O13	72.18
C19 H15 N3 O8		413.09	413.0859	-9.99	C19 H16 N3 O8	66.38
C13 H15 N7 O9		413.0901	413.0931	7.44	C13 H16 N7 O9	64.56

--- End Of Report ---

Figure S 38 MS spectra of compound **3h**

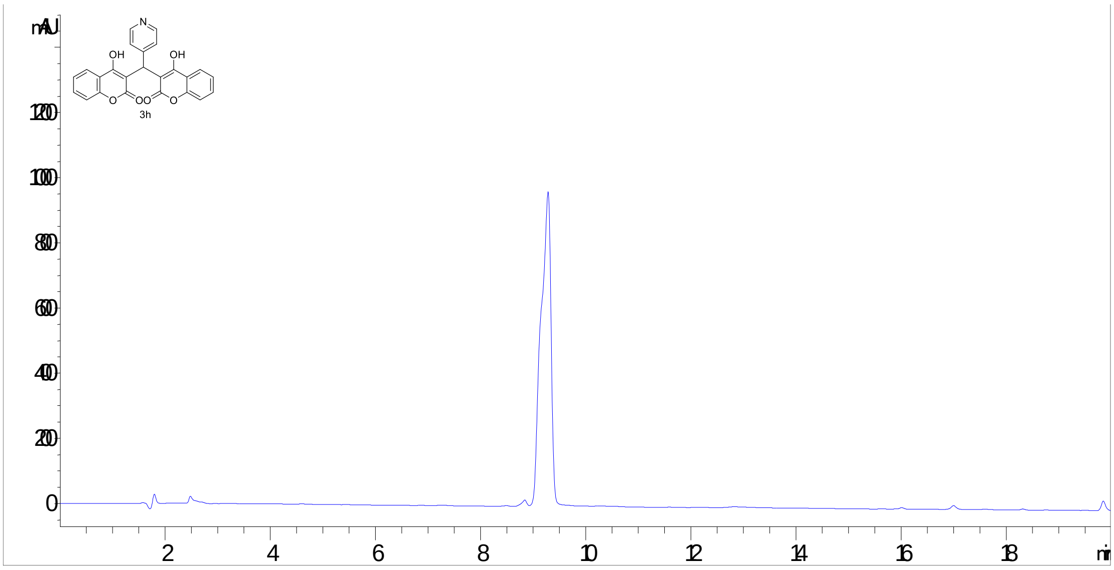

Figure S 39 The purity of **3h** from HPLC

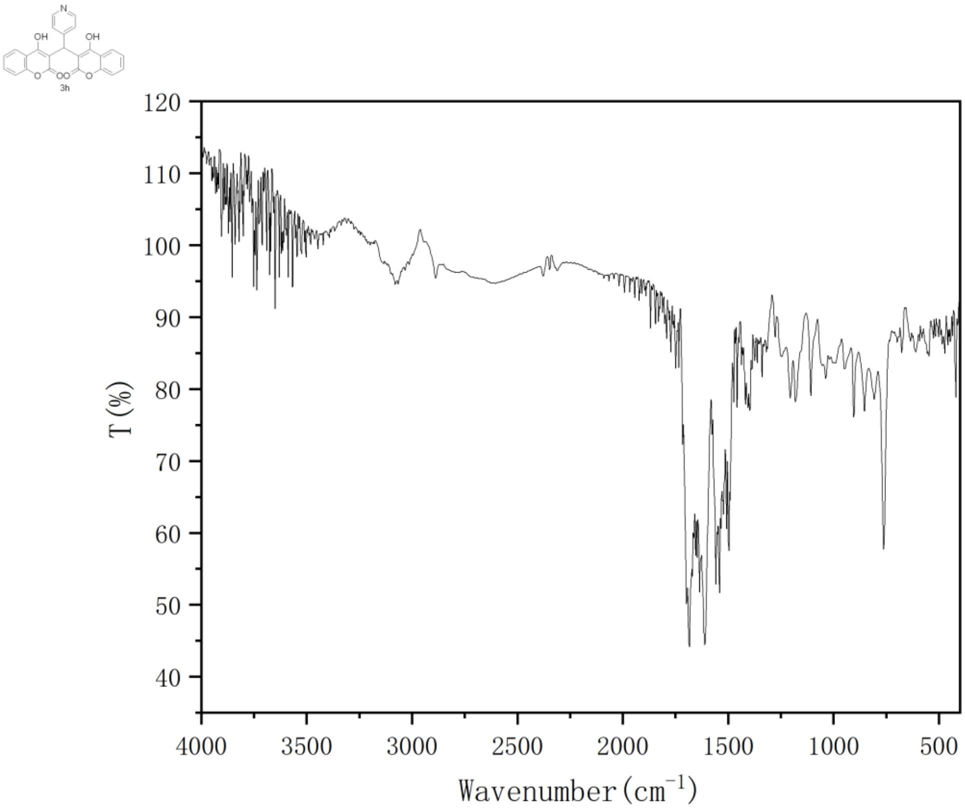

Figure S 40 The purity of **3h** from FTIR

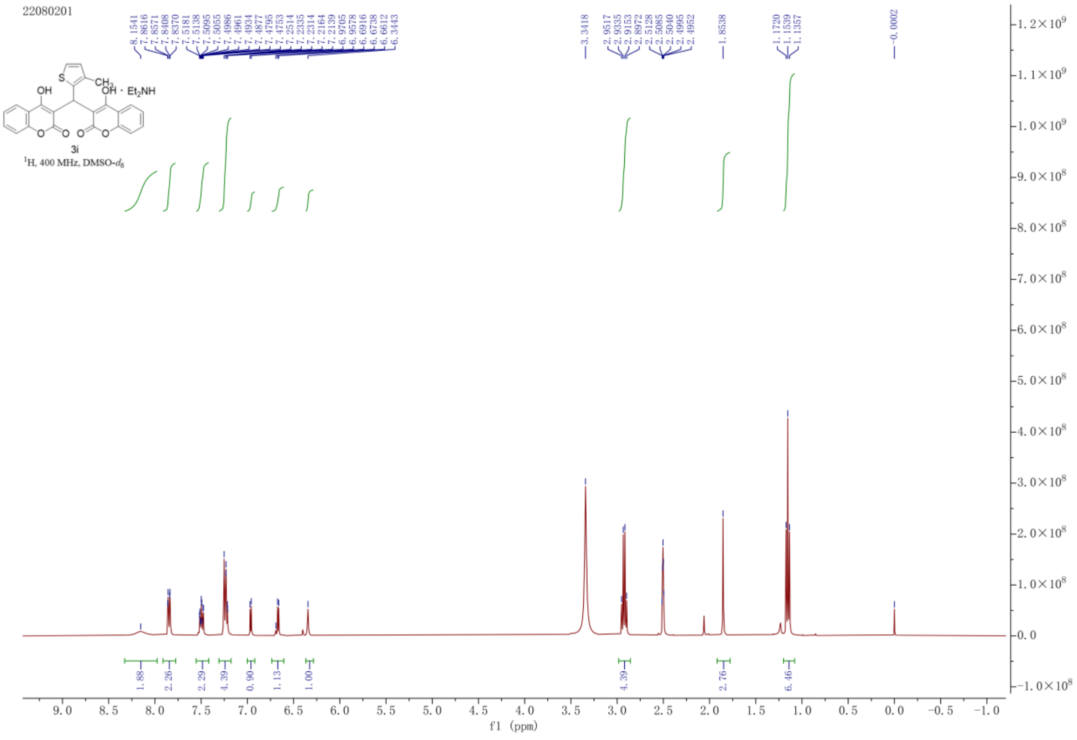

Figure S 41 ^{1}H NMR spectra of compound **3i**

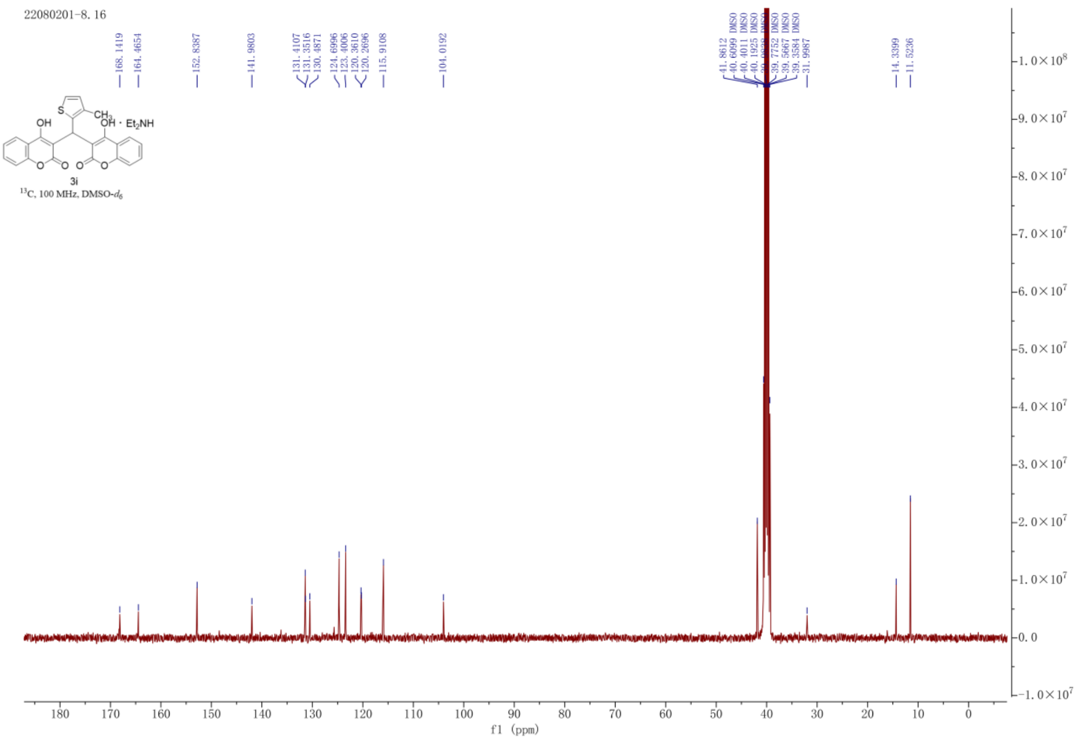

Figure S 42 ^{13}C NMR spectra of compound **3i**

Qualitative Analysis Report

**Data Filename**	21-Dicoumarolum-10.d	**Sample Name**	ZDJ
**Sample Type**	Sample	**Position**	P1-B1
**Instrument Name**	Instrument 1	**User Name**	
**Acq Method**	test.m	**Acquired Time**	11/6/2023 4:38:21 PM
**IRM Calibration Status**	Success	**DA Method**	Default.m
**Comment**			

Sample Group **Info.**

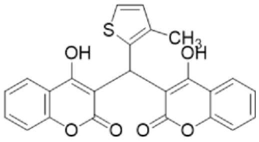

Chemical Formula: $C_{24}H_{16}O_6S$
Exact Mass: 432.0668

User Spectra

Fragmentor Voltage	Collision Energy	Ionization Mode
180	0	ESI

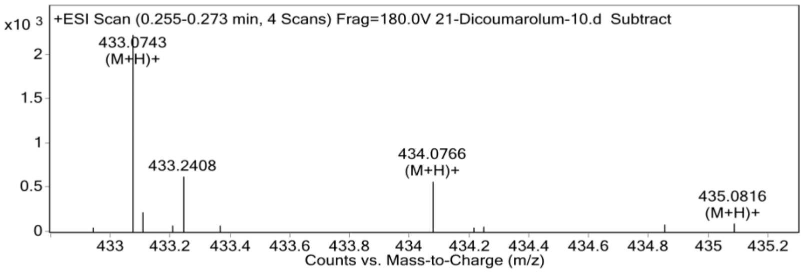

Formula Calculator Element Limits

Element	Min	Max
C	3	60
H	0	120
O	0	30
S	0	10

Formula Calculator Results

Formula	Best	Mass	Tgt Mass	Diff (ppm)	Ion Species	Score
C24 H16 O6 S	TRUE	432.067	432.0668	-0.66	C24 H17 O6 S	90.18
C20 H16 O11		432.067	432.0693	5.14	C20 H17 O11	84.89
C27 H12 O6		432.067	432.0634	-8.45	C27 H13 O6	73.05
C25 H20 O S3		432.067	432.0676	1.34	C25 H21 O S3	72.13
C28 H16 O S2		432.067	432.0643	-6.45	C28 H17 O S2	64.07

--- End Of Report ---

Figure S 43 MS spectra of compound **3i**

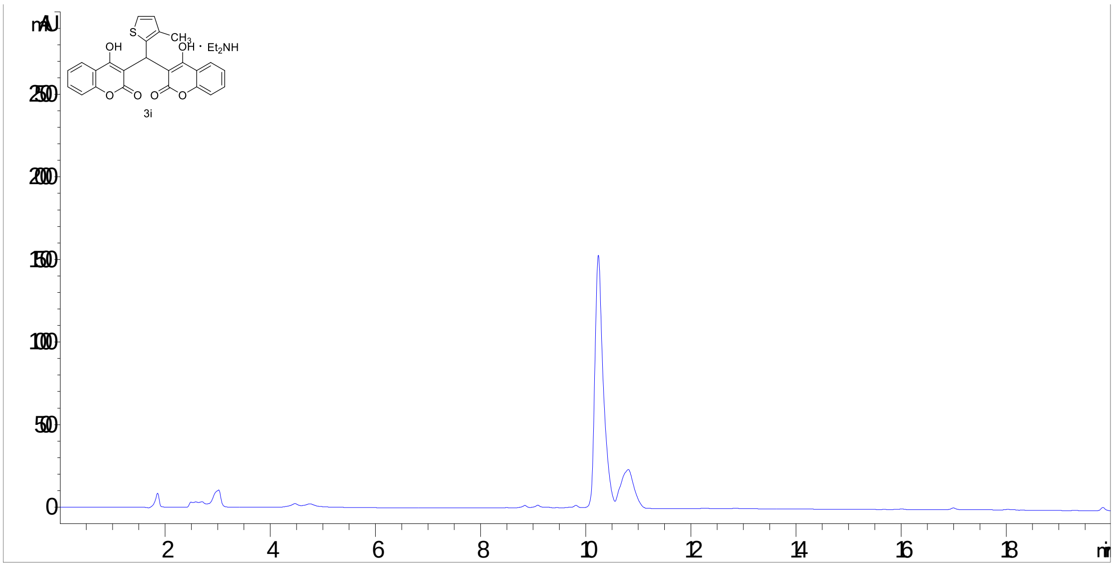

Figure S 44 The purity of **3i** from HPLC

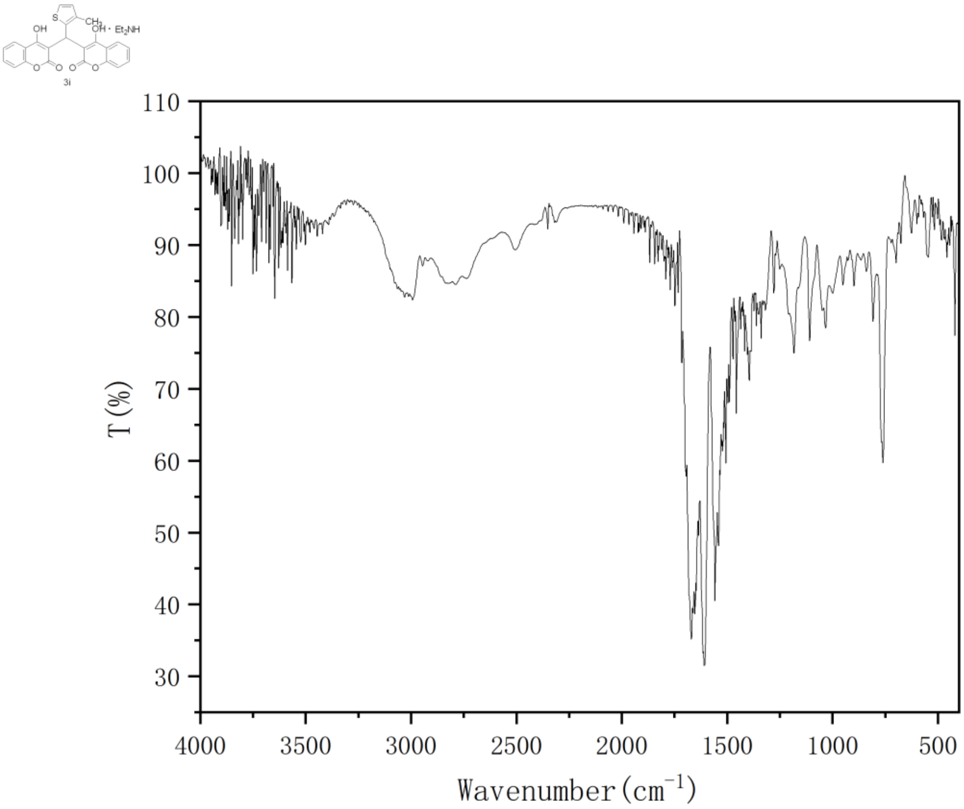

Figure S 45 The purity of **3i** from FTIR

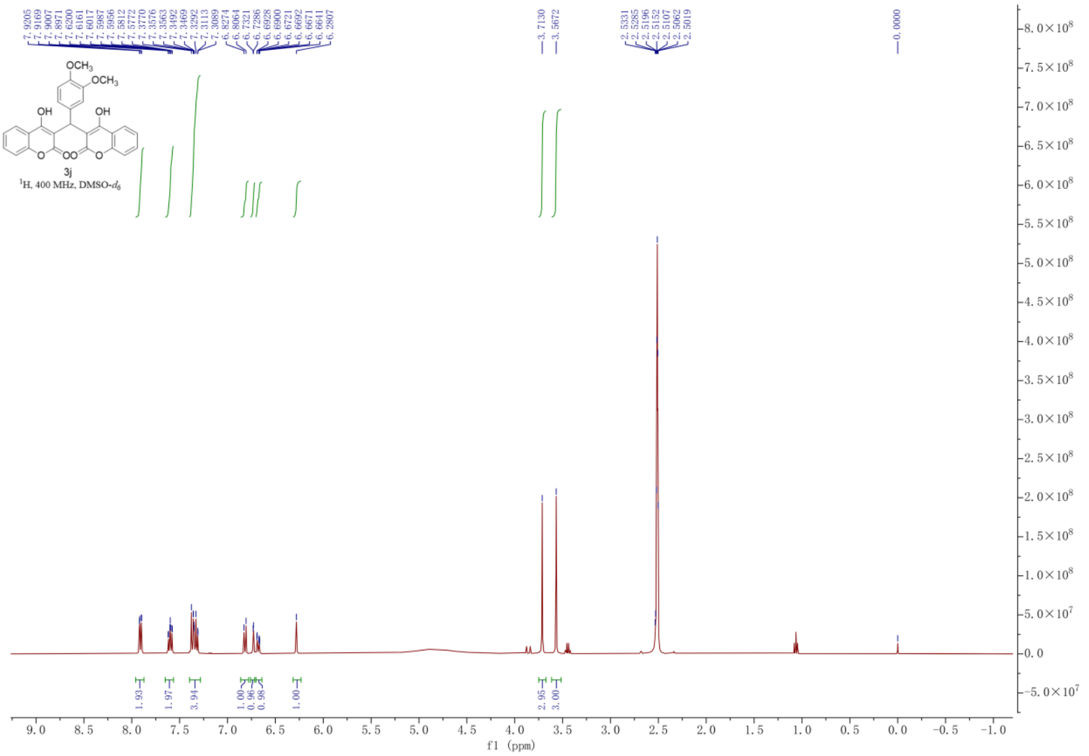

Figure S 46 ^{1}H NMR spectra of compound **3j**

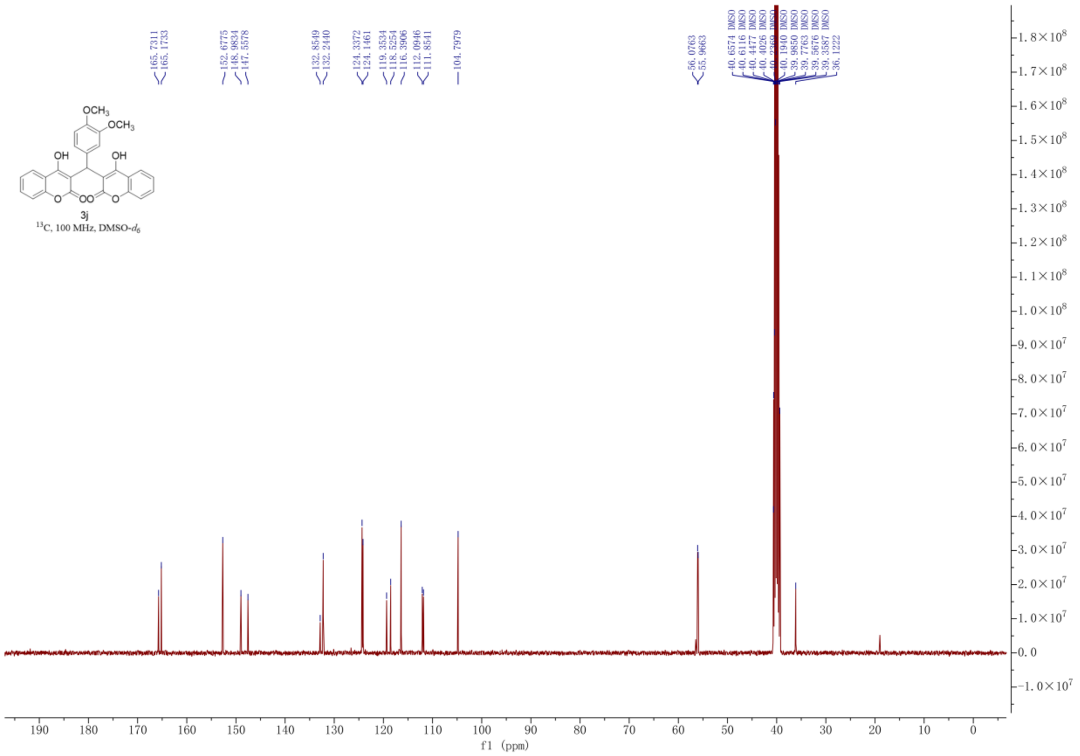

Figure S 47 ^{13}C NMR spectra of compound **3j**

Qualitative Analysis Report

**Data Filename**	22-Dicoumarolum-11.d	**Sample Name**	ZDJ
**Sample Type**	Sample	**Position**	P1-B2
**Instrument Name**	Instrument 1	**User Name**	
**Acq Method**	test.m	**Acquired Time**	11/6/2023 4:39:33 PM
**IRM Calibration Status**	Success	**DA Method**	Default.m
**Comment**			

Sample Group **Info.**

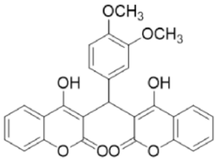

Chemical Formula: $C_{27}H_{20}O_8$
Exact Mass: 472.1158

User Spectra

Fragmentor Voltage	Collision Energy	Ionization Mode
180	0	ESI

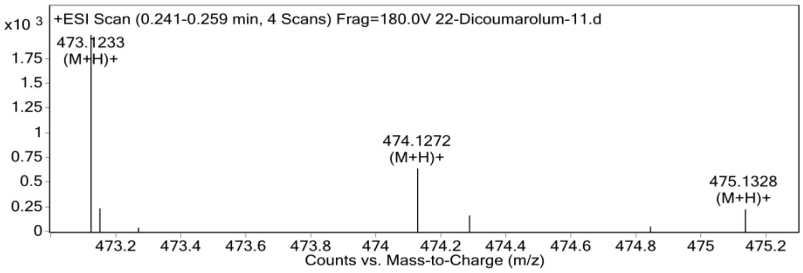

Formula Calculator Element Limits

Element	Min	Max
C	3	60
H	0	120
O	0	30

Formula Calculator Results

Formula	Best	Mass	Tgt Mass	Diff (ppm)	Ion Species	Score
C27 H20 O8	TRUE	472.116	472.1158	-0.39	C27 H21 O8	88.65

--- End Of Report ---

Figure S 48 MS spectra of compound **3j**

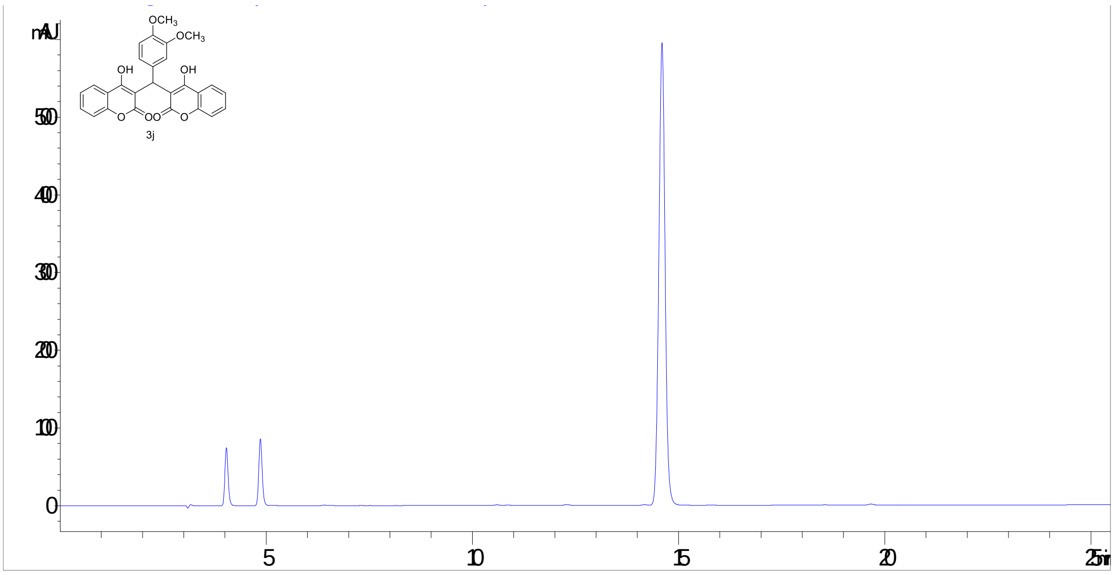

Figure S 49 The purity of **3j** from HPLC

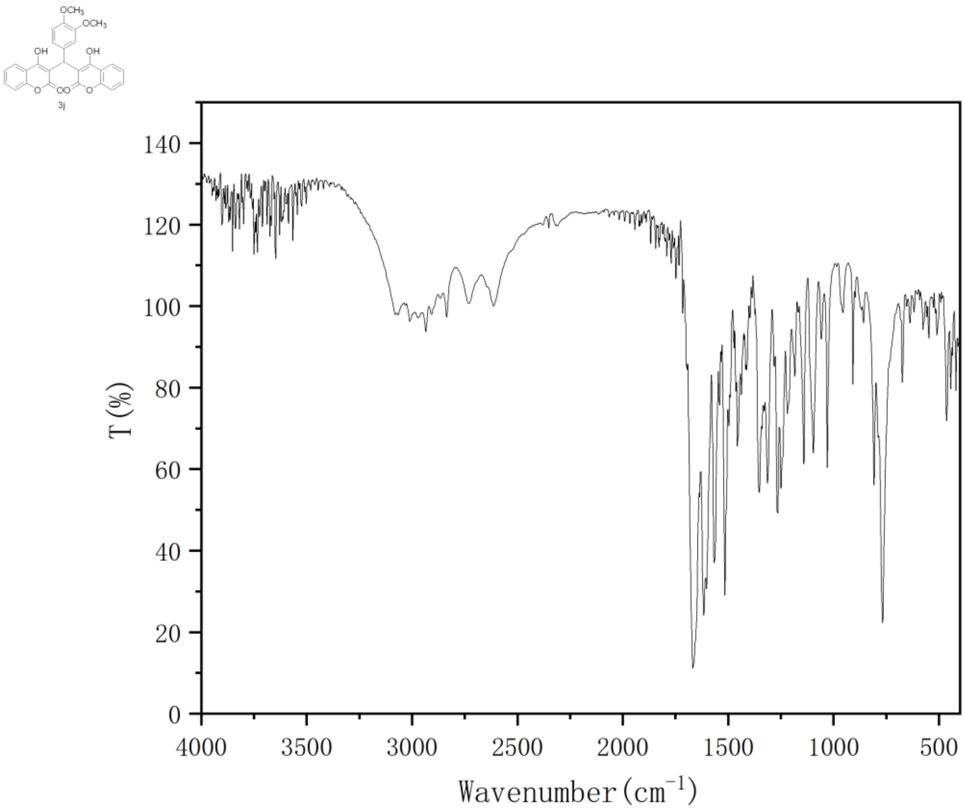

Figure S 50 The purity of **3j** from FTIR

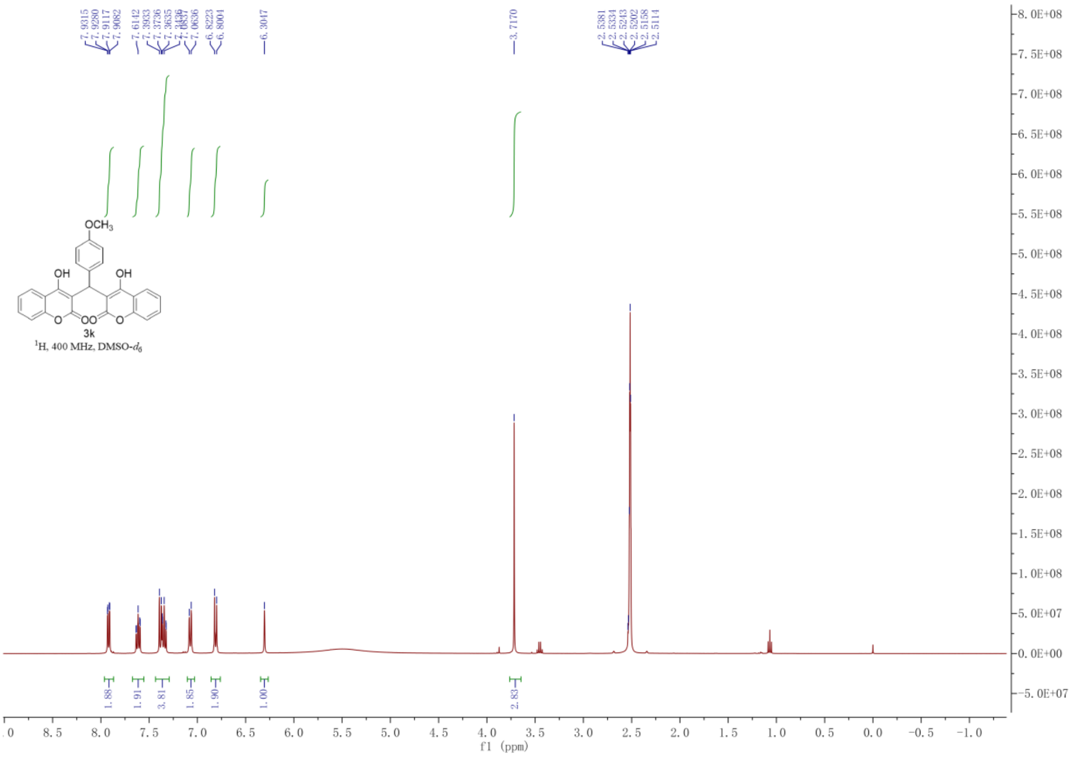

Figure S 51 ^{1}H NMR spectra of compound **3k**

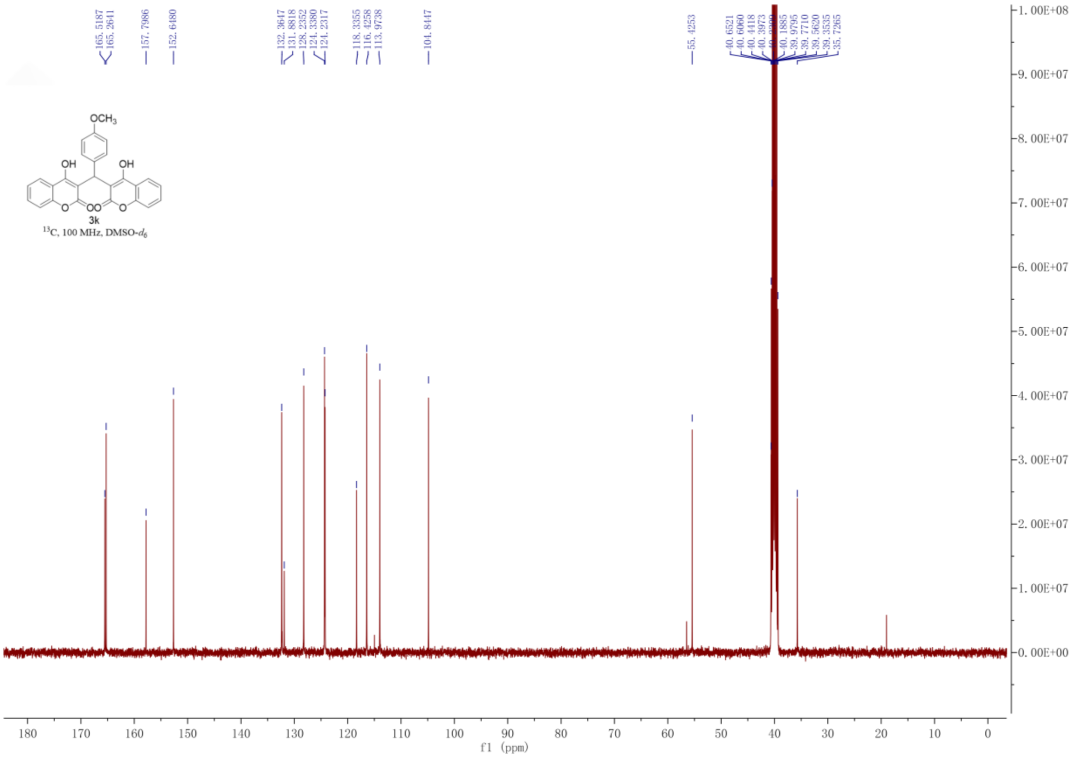

Figure S 52 ^{13}C NMR spectra of compound **3k**

Qualitative Analysis Report

**Data Filename**	23-Dicoumarolum-12.d	**Sample Name**	ZDJ
**Sample Type**	Sample	**Position**	P1-B3
**Instrument Name**	Instrument 1	**User Name**	
**Acq Method**	test.m	**Acquired Time**	11/6/2023 4:40:46 PM
**IRM Calibration Status**	Success	**DA Method**	Default.m
**Comment**			

Sample Group **Info.**

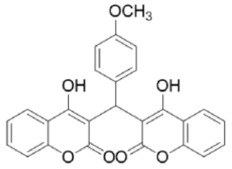

Chemical Formula: $C_{26}H_{18}O_7$
Exact Mass: 442.1053

User Spectra

Fragmentor Voltage	Collision Energy	Ionization Mode
180	0	ESI

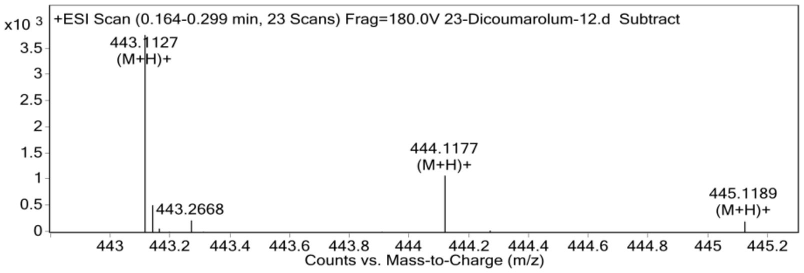

Formula Calculator Element Limits

Element	Min	Max
C	3	60
H	0	120
O	0	30

Formula Calculator Results

Formula	Best	Mass	Tgt Mass	Diff (ppm)	Ion Species	Score
C26 H18 O7	TRUE	442.1055	442.1053	-0.5	C26 H19 O7	97.39

--- End Of Report ---

Figure S 53 MS spectra of compound **3k**

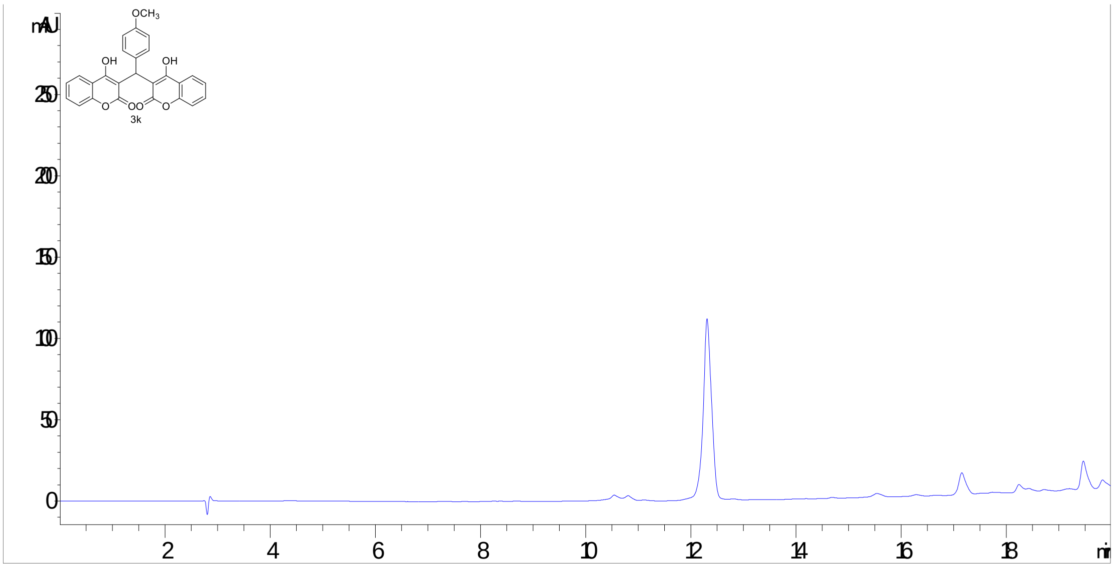

Figure S 54 The purity of **3k** from HPLC

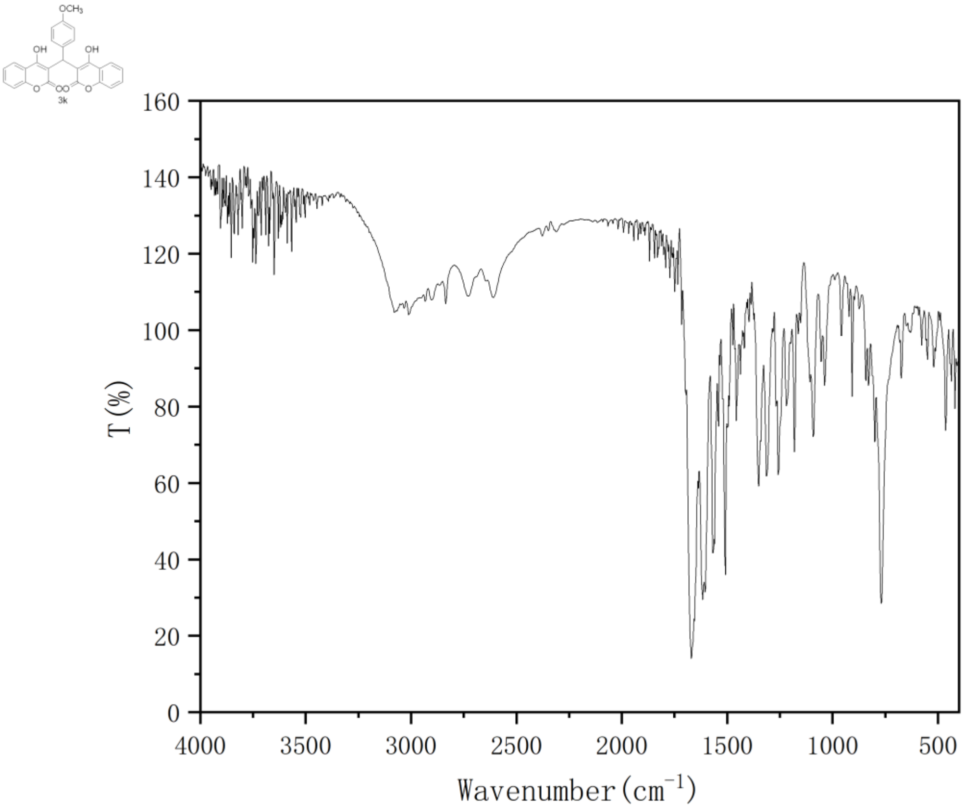

Figure S 55 The purity of **3k** from FTIR

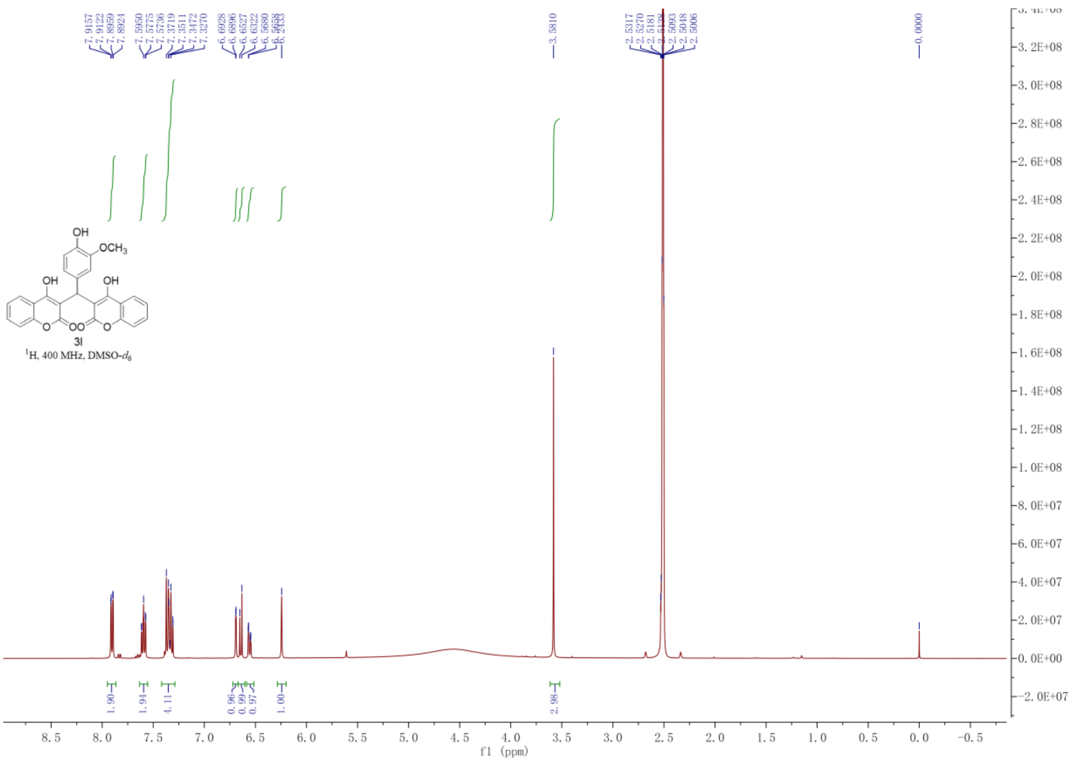

Figure S 56 ^{1}H NMR spectra of compound **3l**

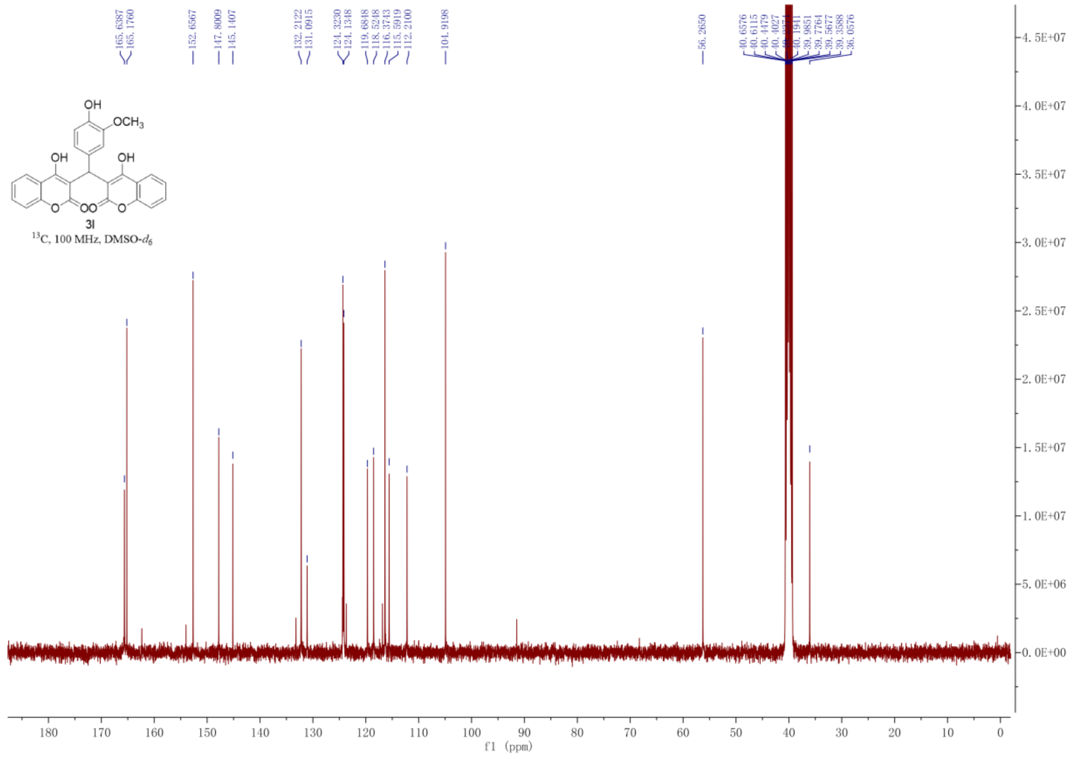

Figure S 57 ^{13}C NMR spectra of compound **3l**

Qualitative Analysis Report

**Data Filename**	24-Dicoumarolum-13.d	**Sample Name**	ZDJ
**Sample Type**	Sample	**Position**	P1-B4
**Instrument Name**	Instrument 1	**User Name**	
**Acq Method**	test.m	**Acquired Time**	11/6/2023 4:41:59 PM
**IRM Calibration Status**	Success	**DA Method**	Default.m
**Comment**			

Sample Group **Info.**

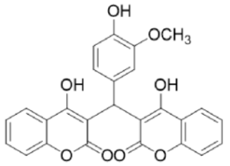

Chemical Formula: $C_{26}H_{18}O_8$
Exact Mass: 458.1002

User Spectra

Fragmentor Voltage	Collision Energy	Ionization Mode
180	0	ESI

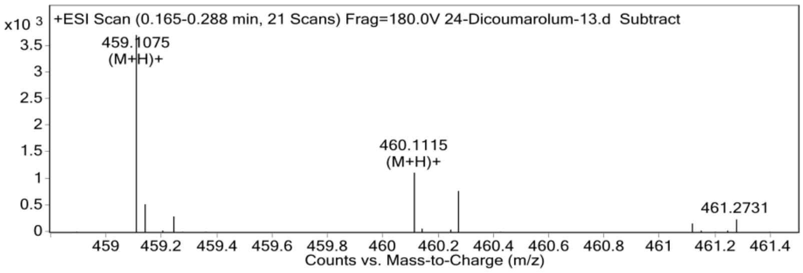

Formula Calculator Element Limits

Element	Min	Max
C	3	60
H	0	120
O	0	30

Formula Calculator Results

Formula	Best	Mass	Tgt Mass	Diff (ppm)	Ion Species	Score
C26 H18 O8	TRUE	458.1003	458.1002	-0.23	C26 H19 O8	98.21

--- End Of Report ---

Figure S 58 MS spectra of compound **3l**

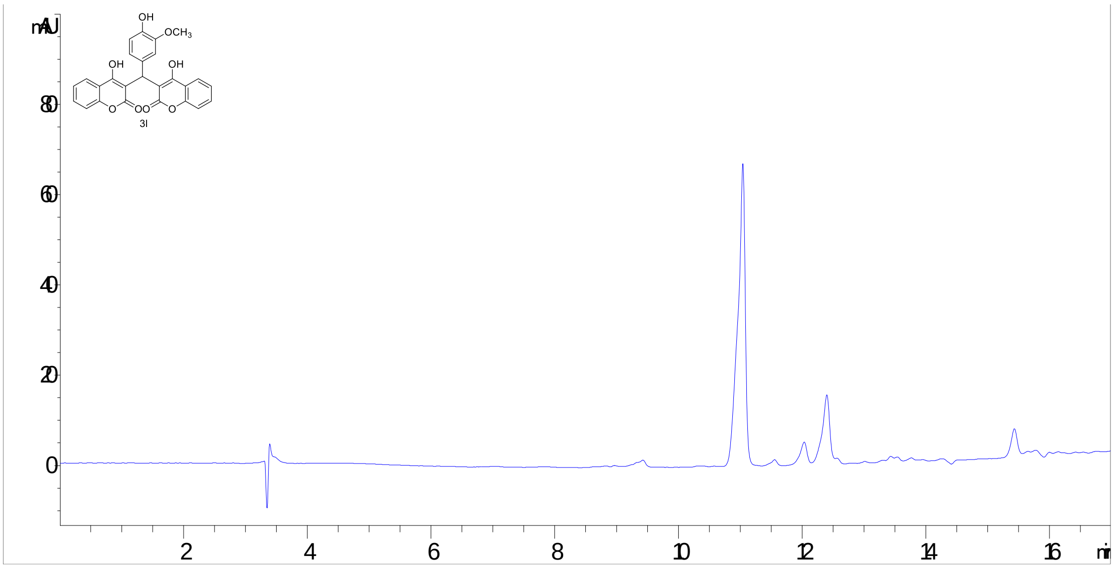

Figure S 59 The purity of **3l** from HPLC

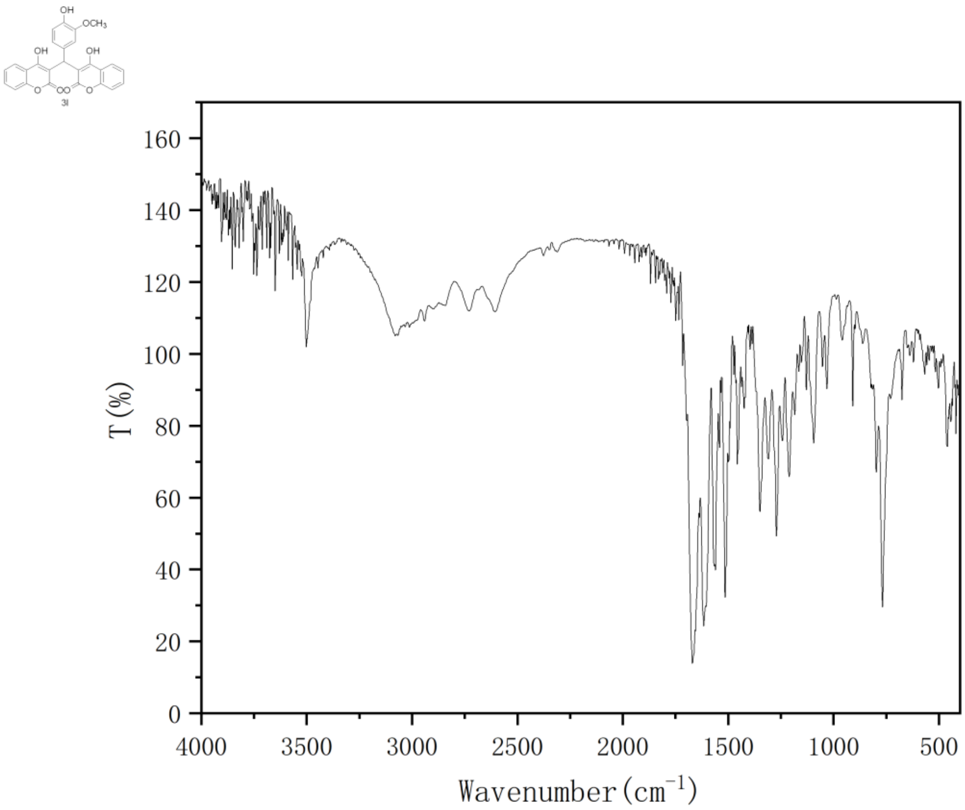

Figure S 60 The purity of **3l** from FTIR

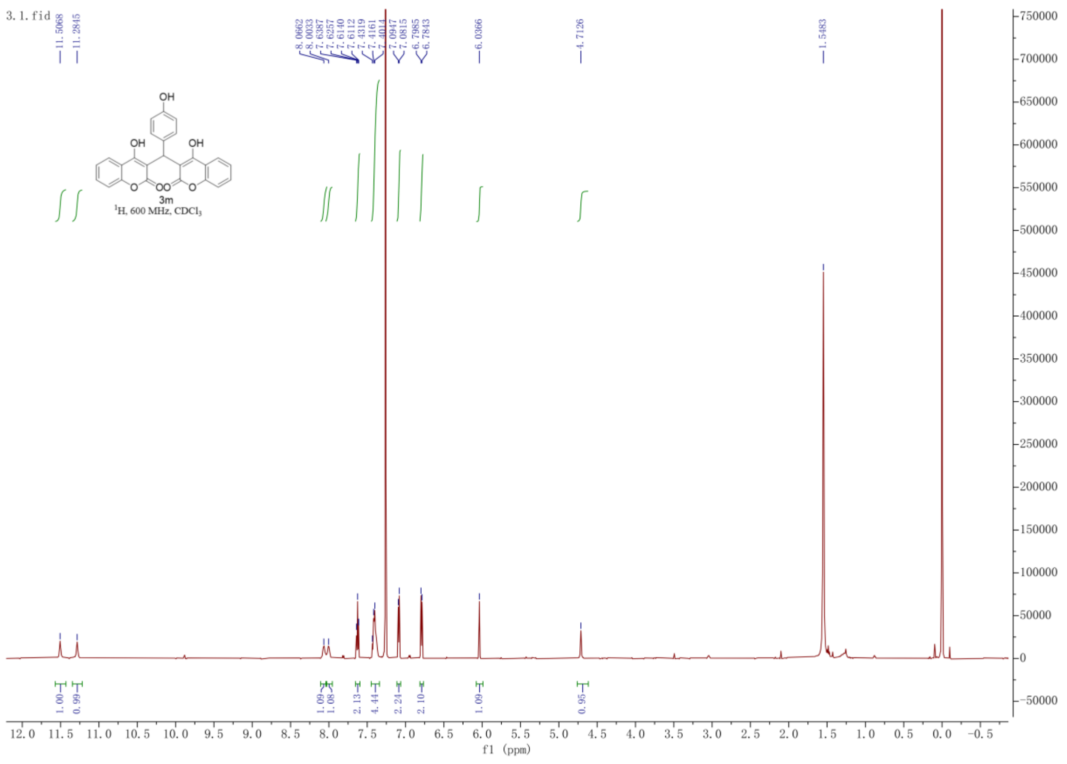

Figure S 61 ^{1}H NMR spectra of compound **3m**

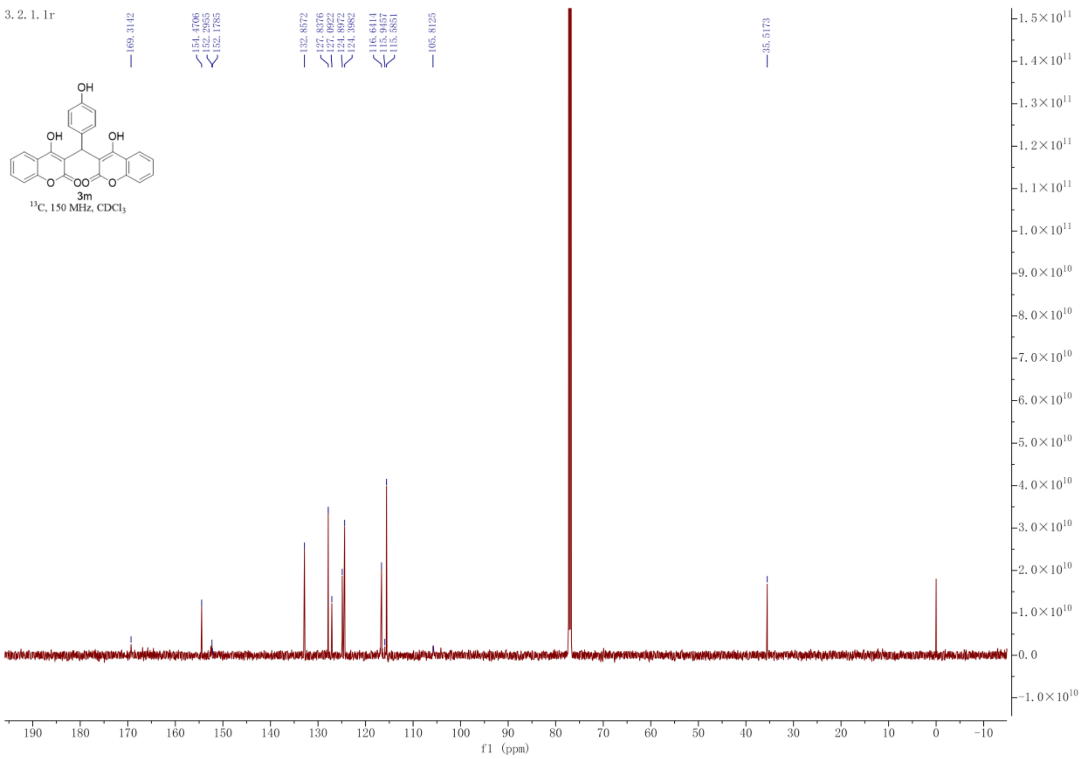

Figure S 62 ^{13}C NMR spectra of compound **3m**

Qualitative Analysis Report

**Data Filename**	25-Dicoumarolum-14.d	**Sample Name**	ZDJ
**Sample Type**	Sample	**Position**	P1-B5
**Instrument Name**	Instrument 1	**User Name**	
**Acq Method**	test.m	**Acquired Time**	11/6/2023 4:43:11 PM
**IRM Calibration Status**	Success	**DA Method**	Default.m
**Comment**			

Sample Group **Info.**

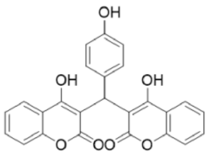

Chemical Formula: $C_{25}H_{16}O_7$
Exact Mass: 428.0896

User Spectra

Fragmentor Voltage	Collision Energy	Ionization Mode
180	0	ESI

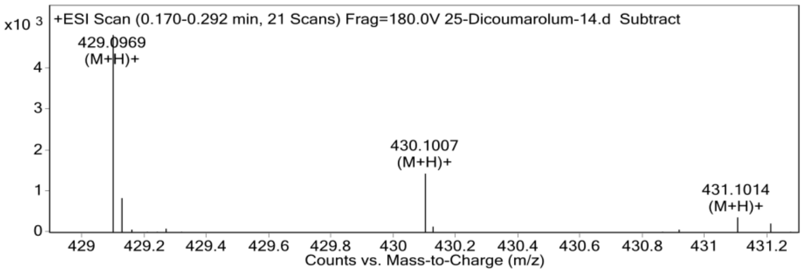

Formula Calculator Element Limits

Element	Min	Max
C	3	60
H	0	120
O	0	30

Formula Calculator Results

Formula	Best	Mass	Tgt Mass	Diff (ppm)	Ion Species	Score
C25 H16 O7	TRUE	428.0896	428.0896	-0.05	C25 H17 O7	97.03

--- End Of Report ---

Figure S 63 MS spectra of compound **3m**

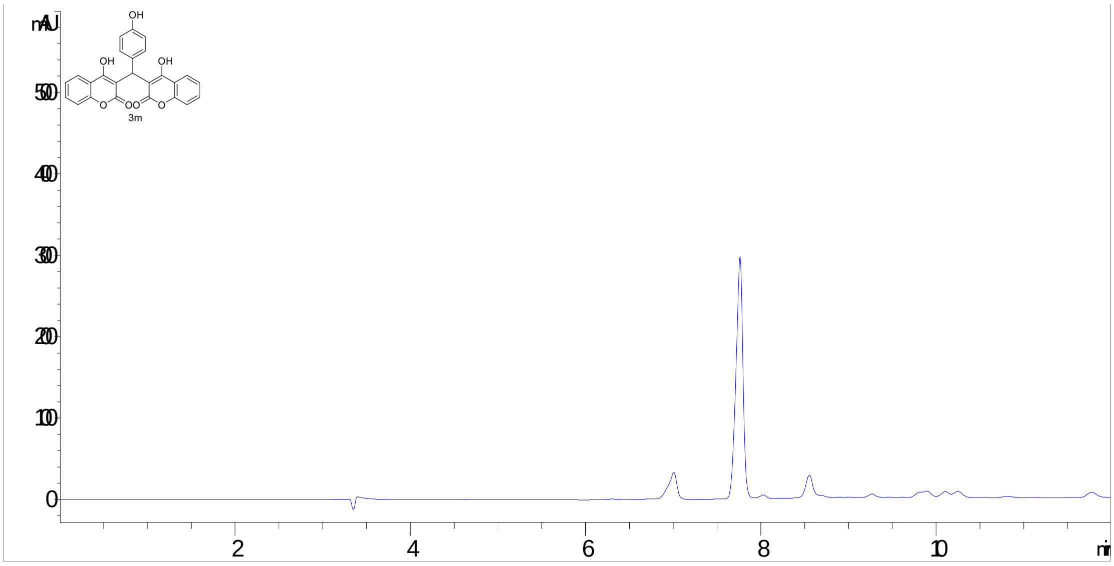

Figure S 64 The purity of **3m** from HPLC

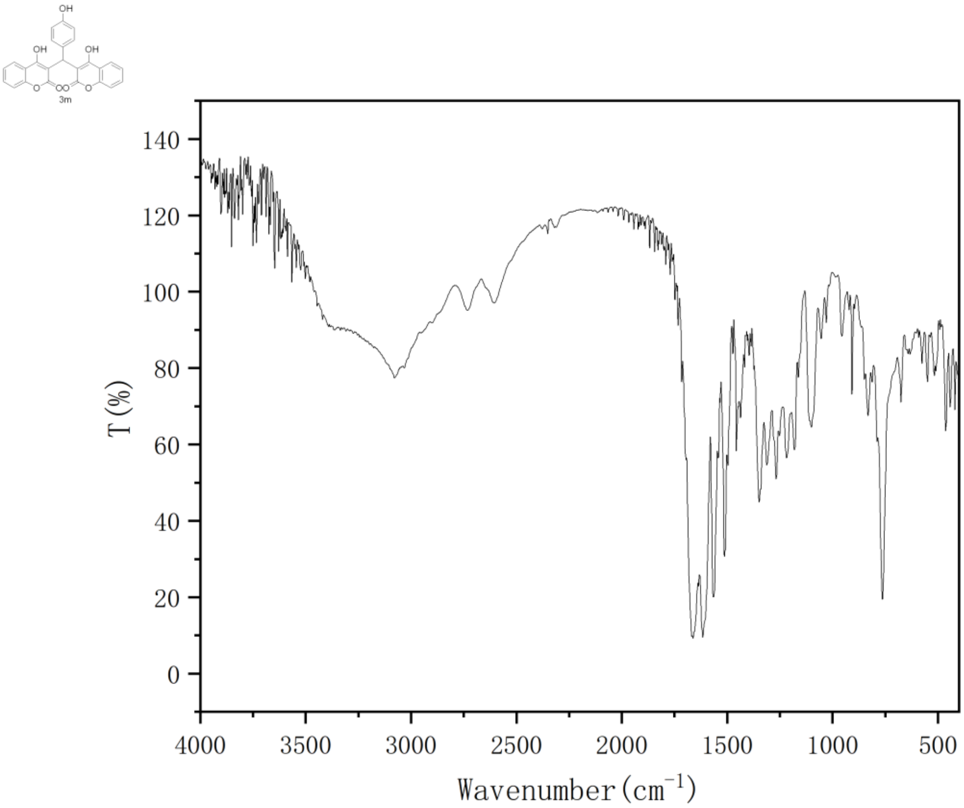

Figure S 65 The purity of **3m** from FTIR

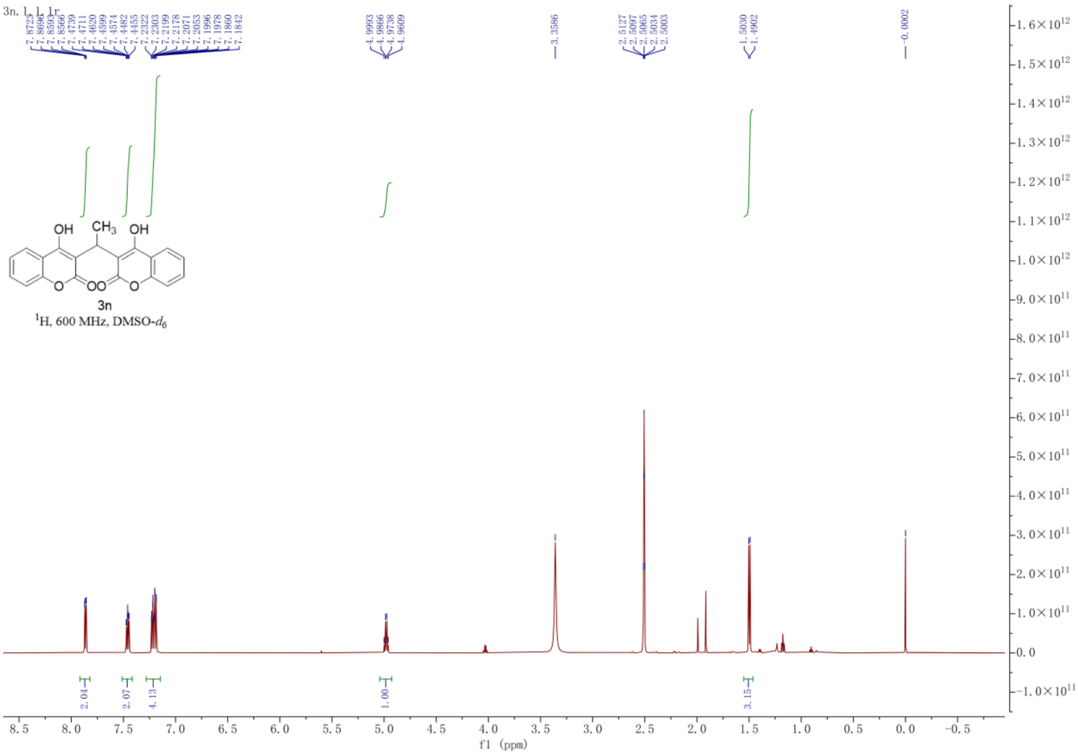

Figure S 66 ^{1}H NMR spectra of compound **3n**

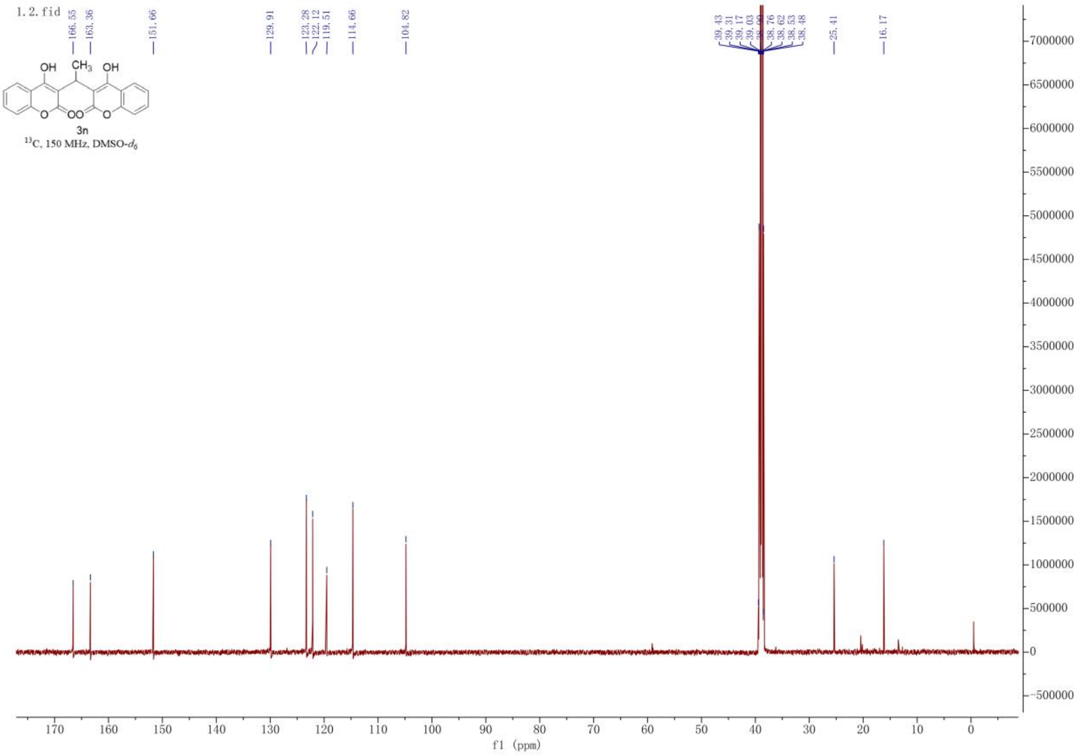

Figure S 67 ^{13}C NMR spectra of compound **3n**

Qualitative Analysis Report

**Data Filename**	26-Dicoumarolum-15.d	**Sample Name**	ZDJ
**Sample Type**	Sample	**Position**	P1-B6
**Instrument Name**	Instrument 1	**User Name**	
**Acq Method**	test.m	**Acquired Time**	11/6/2023 5:17:27 PM
**IRM Calibration Status**	Success	**DA Method**	Default.m
**Comment**			

Sample Group **Info.**

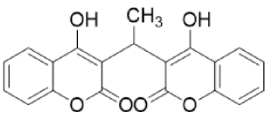

Chemical Formula: $C_{20}H_{14}O_6$
Exact Mass: 350.0790

User Spectra

Fragmentor Voltage	Collision Energy	Ionization Mode
180	0	ESI

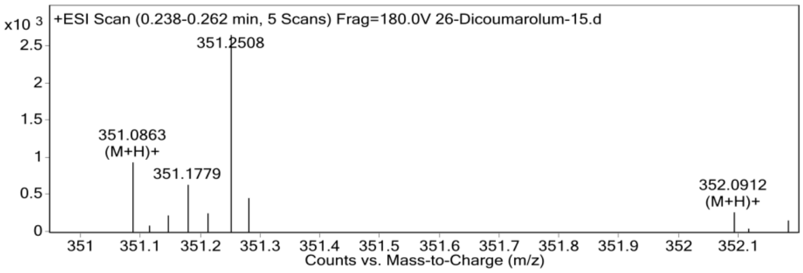

Formula Calculator Element Limits

Element	Min	Max
C	3	60
H	0	120
O	0	30

Formula Calculator Results

Formula	Best	Mass	Tgt Mass	Diff (ppm)	Ion Species	Score
C20 H14 O6	TRUE	350.079	350.079	0.02	C20 H15 O6	83.51

--- End Of Report ---

Figure S 68 MS spectra of compound **3n**

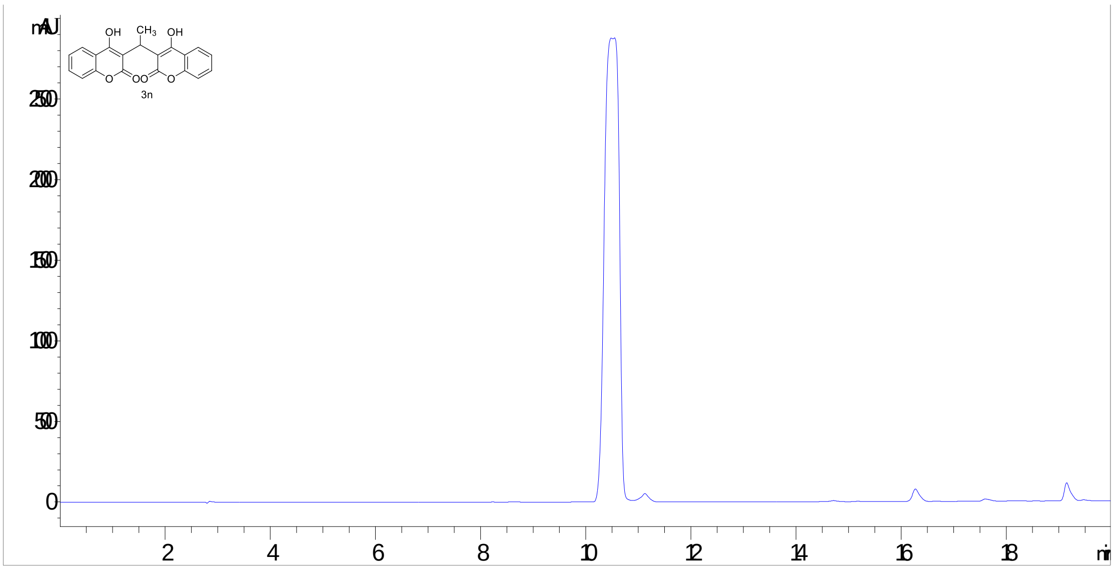

Figure S 69 The purity of **3n** from HPLC

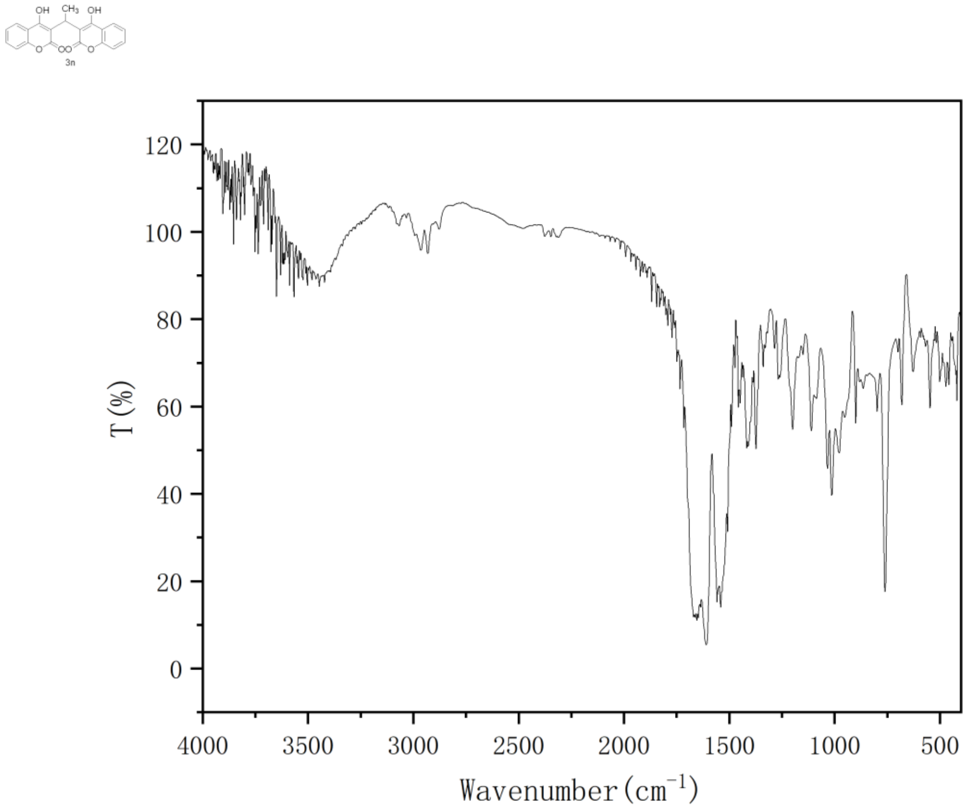

Figure S 70 The purity of **3n** from FTIR
